# *K*-mer-based Approaches to Bridging Pangenomics and Population Genetics

**DOI:** 10.1093/molbev/msaf047

**Published:** 2025-03-05

**Authors:** Miles D Roberts, Olivia Davis, Emily B Josephs, Robert J Williamson

**Affiliations:** Genetics and Genome Sciences Program, Michigan State University, East Lansing, MI 48824, USA; Department of Computer Science and Software Engineering, Rose-Hulman Institute of Technology, Terre Haute, IN 47803, USA; Department of Plant Biology, Michigan State University, East Lansing, MI 48824, USA; Ecology, Evolution, and Behavior Program, Michigan State University, East Lansing, MI 48824, USA; Plant Resilience Institute, Michigan State University, East Lansing, MI 48824, USA; Department of Computer Science and Software Engineering, Rose-Hulman Institute of Technology, Terre Haute, IN 47803, USA; Department of Biology and Biomedical Engineering, Rose-Hulman Institute of Technology, Terre Haute, IN 47803, USA

**Keywords:** population genomics, *k*-mer, bloom filter, pangenomics

## Abstract

Many commonly studied species now have more than one chromosome-scale genome assembly, revealing a large amount of genetic diversity previously missed by approaches that map short reads to a single reference. However, many species still lack multiple reference genomes and correctly aligning references to build pangenomes can be challenging for many species, limiting our ability to study this missing genomic variation in population genetics. Here, we argue that *k*-mers are a very useful but underutilized tool for bridging the reference-focused paradigms of population genetics with the reference-free paradigms of pangenomics. We review current literature on the uses of *k*-mers for performing three core components of most population genetics analyses: identifying, measuring, and explaining patterns of genetic variation. We also demonstrate how different *k*-mer-based measures of genetic variation behave in population genetic simulations according to the choice of *k*, depth of sequencing coverage, and degree of data compression. Overall, we find that *k*-mer-based measures of genetic diversity scale consistently with pairwise nucleotide diversity (*π*) up to values of about π=0.025 ($R^{2}=0.97$) for neutrally evolving populations. For populations with even more variation, using shorter *k*-mers will maintain the scalability up to at least π=0.1. Furthermore, in our simulated populations, *k*-mer dissimilarity values can be reliably approximated from counting bloom filters, highlighting a potential avenue to decreasing the memory burden of *k*-mer-based genomic dissimilarity analyses. For future studies, there is a great opportunity to further develop methods to identifying selected loci using *k*-mers.

## Introduction

Two decades ago, assembling one reference genome for one eukaryotic species was an international, herculean effort (Venter et al. 2001). Now, individual laboratories can readily assemble and align multiple reference-quality genomes from the same species into pangenomes (Golicz et al. 2020). This shift toward pangenomes as the basis for genetic studies is already transforming our understanding of genetic variation in populations. Analysis of pangenomes has uncovered vast quantities of genetic variation previously missed by the ubiquitous practice of aligning short reads to a single reference genome (Wong et al. 2020; Sirén et al. 2021; Ebler et al. 2022; Zhou et al. 2022; Liao et al. 2023; Rice et al. 2023), increased the power of trait mapping (Song et al. 2020; Chin et al. 2023), and resolved complex structural variations (Hickey et al. 2020; Song et al. 2024). Pangenomes have even revised our understanding of previously cataloged variation, because single nucleotide polymorphisms (SNPs) once identified by mapping reads against a single reference can sometimes be resolved as alignment errors due to structural variation (Jaegle et al. 2023). Altogether, pangenomes provide a more accurate representation of genetic variation, reducing the commonly observed phenomenon where one’s choice of reference genome shapes the ultimate conclusions of a study (i.e. reference bias, Gage et al. 2019; Günther and Nettelblad 2019; Chen et al. 2021; Ebler et al. 2022). This better ability to capture and explain patterns of genetic variation, combined with recent developments in pangenome assembly algorithms (Hickey et al. 2020; Garrison et al. 2024) and an explosion in pangenome sequencing for many nonmodel organisms (Lei et al. 2021), means that pangenomes will likely be the standard for population genetics analysis in the near future.

However, pangenomes can be difficult to assemble and tune in some contexts (Hahn 2018; Song et al. 2024). First, pangenomes by definition require more sequencing data to assemble compared to a single reference genome, which can make building pangenomes expensive for large genome species or study systems with fewer resources. Furthermore, whole genome alignment (WGA) is key for pangenome assembly but also difficult to tune, affecting pangenome analysis. Modern WGA algorithms are impressive and are being used to produce pangenomes for a wide range of species (Garrison et al. 2024); however, current WGA algorithms are mainly developed and tuned to align human and model species genomes, and so do not always generalize well to genomes that are large, highly repetitive, highly diverse, polyploid, or containing high levels of structural variation (reviewed in Song et al. 2024). For example, corn (*Zea mays*) has a famously repetitive and structurally variable genome and new WGA approaches needed to be developed just to properly align corn genomes (Song et al. 2022). There are also still many open questions on how to best represent complex, nested variations in such alignments and tune alignment parameters (Song et al. 2024). A researcher’s exact choice of alignment parameters and software can drastically affect the shape of a pangenome graph (Rice et al. 2023) and downstream genotype calls (O’Rawe et al. 2013; Li 2014; Bush et al. 2020; Betschart et al. 2022; Sopniewski and Catullo 2024). Given the challenges of computing and tuning alignments, approaches that skip alignment altogether could valuably complement or help guide pangenome assembly.

In this review, we argue that the *k*-mers deserve more attention from population geneticists because they can complement the study of pangenomes. A *k*-mer is a sub-sequence of length *k* within a larger sequence. For example, the sequence “ATGCA,” contains the unique 2-mers AT, TG, GC, and CA. The main benefit of *k*-mers is that they can be analyzed without alignment. Instead, one simply counts all of the unique *k*-mers present in a sample of reads (where *k*-mers that are reverse complements of each other are typically considered identical and are counted together Kokot et al. 2017), then uses the resulting count matrix for downstream analysis (see Fig. 2). Thus, *k*-mers derived from regions far diverged, absent, or otherwise unalignable in a given reference will not be automatically excluded from an analysis, allowing one to get a picture of pangenomic variation. *k*-mers have a long history of use in metagenomics (McClelland 1985; Rosen et al. 2008; Dubinkina et al. 2016), phylogenetics (Kolekar et al. 2012; Haubold 2014; Zielezinski et al. 2017, 2019; Bussi et al. 2021; Beichman et al. 2023; Jenike et al. 2025), computer science (Shannon 1948), and quantitative genetics (Kim et al. 2020; Voichek and Weigel 2020; Gupta 2021; Lemane et al. 2022; Onetto et al. 2022). However, while applications of *k*-mers receive much attention in other disciplines, population genetic investigations using *k*-mers remain limited.

Our goal is to review *k*-mer-based approaches for identifying, measuring, and explaining patterns of genetic variation in populations. At the same time, we investigate the behavior of *k*-mer-based measures of variation to the choice of *k*, the depth of sequencing coverage, and the degree of data compression. We finally highlight some avenues to explore in *k*-mer-based (i.e. reference-free or alignment-free) population genetics. Overall, we advocate that *k*-mer-based approaches can be valuable complements to common reference-based population genetics methods.

## Box 1: What is the “Best” Value for *k*?

A common first question in *k*-mer-based analyses is: What value(s) of *k* should be analyzed? The “best” *k* for an analysis is ultimately determined by a trade-off between the length of *k* and sequencing error: longer *k*-mers are more likely to represent unique genomic sequences, but are also more likely to contain a sequencing error (Rahman et al. 2018). In practice, many studies use a *k* of around 20–40 bp (Ponsero et al. 2023) because *k*-mers in this range can be reliably sequenced with short read data and often align uniquely to their source genome (Wu et al. 1991; Becher et al. 2022). For example, k=32 captures 85.7% of unique sequences in the human genome (Shajii et al. 2016), while k=21 distinguishes many eukaryote, bacteria, and archea species (Bussi et al. 2021). However, there are many past studies that propose criteria for choosing specific values of *k*.

### Choosing Multiple Values of *k*

One “brute force” approach to test the sensitivity of results to *k* is to simply repeat an analysis multiple times for different values of *k*. This approach is especially common among genome assembly algorithms (Chikhi and Medvedev 2014; Durai and Schulz 2016) but could be applied to almost any analysis in theory. The main drawback, however, is the high computational burden of performing the same analysis multiple times. It is also difficult to know without more information whether analyses performed for certain values of *k* produce more accurate results than analyses for other values of *k*, motivating the need for *k* selection criteria that can be either minimized or maximized.

### Choosing *k* Based on the Number of Unique Nonerroneous *k*-mers

Higher values of *k* generally allow greater detection of unique sequences but increase the probability of observing *k*-mers containing at least one sequencing error (Chikhi and Medvedev 2014; Rahman et al. 2018). Each sequencing error can result in up to *k* erroneous *k*-mers, making it important to prevent errors from dominating one’s analysis. Thus, choosing a *k* that maximizes the number of unique nonerroneous *k*-mers in a dataset is generally considered optimal for tasks like genome assembly (Chikhi and Medvedev 2014). This approach generally involves measuring the *k*-mer frequency spectrum and then fitting a model to the distribution to estimate which parts of the spectrum come from erroneous *k*-mers (Chikhi and Medvedev 2014). Usually, the low-frequency end of the spectrum is dominated by erroneous *k*-mers because sequencing errors are unlikely to generate the same erroneous *k*-mers many times and usually convert real *k*-mers into *k*-mers not found in the source genome (Kelley et al. 2010). Although this criterion for choosing *k* could be applied to population genetic datasets, it is not if clear the resulting optimal *k* would vary significantly between genomes within the same species. Presumably, if genomes within the same species have considerable variation in repetitive content (Haberer et al. 2020), size (Schmuths et al. 2004), or ploidy (reviewed in Kolář et al. 2017) then the optimal *k* could vary. Determining the value of *k* that maximizes the number of unique nonerroneous *k*-mers across all individuals in a population may be of interest for future population genetics studies.

### Choosing *k* Based on the Probability of Chance *k*-mer Matches Between Samples

Another way to choose *k* is to think about the probability that a *k*-mer from one genome is also found in a second genome by chance alone. For example, it would be unsurprising for almost any pair of reasonably long, naturally occurring DNA sequences to share the *k*-mer “AGC” because this *k*-mer is very short and could occur many times in a random sequence. How large must *k* be then before finding a *k*-mer in two different sequences is unlikely by chance alone? To discuss this question, we next present equations similar to ones in Ondov et al. (2016), except we generalize to account for variation in base composition between sequences being compared.

Let’s begin by imagining we have two genome sequences we wish to compare: $X_{1}$ and $X_{2}$. First, to generate $X_{1}$, we sample with replacement the letters A, T, G, and C a total of *L* times with probabilities $p_{A1}$, $p_{T1}$, $p_{G1}$, and $p_{C1}$, respectively, where $p_{A1}+p_{T1}+p_{G1}+p_{C1}=1$. In other words, we sample our DNA from a multinomial distribution where we assume that each base is sampled independently of the preceding bases. This gives us one strand for $X_{1}$ and we can then generate the complementary strand by pairing A with T and G with C. We repeat this whole process once more to construct $X_{2}$ using probabilities $p_{A2}$, $p_{T2}$, $p_{G2}$, and $p_{C2}$, which may or may not match the probabilities for $X_{1}$, except we only sample *k* bases. This gives us a *k*-mer, *K*, in the genome sequence of $X_{2}$. We now wish to find the probability that *K* occurs in $X_{1}$, assuming that the sampling of bases for *K* was independent of sampling bases for $X_{1}$.

If we imagine constructing $X_{1}$ and *K* at the same time, then the probability of drawing the same letter for both sequences at a given position is:


(1) $$\Sigma_{F}=p_{A1}p_{A2}+p_{T1}p_{T2}+p_{G1}p_{G2}+p_{C1}p_{C2}.$$

However, we also want to consider sequences that are reverse complements as identical, so the probability of drawing a pair of bases that match as reverse complements is:


(2) $$\Sigma_{R}=p_{A1}p_{T2}+p_{T1}p_{A2}+p_{G1}p_{C2}+p_{C1}p_{G2}.$$

The probability of drawing *k* pairs of bases in a row that are either identical or reverse complement matches is thus $\Sigma_{F}^{k}$ and $\Sigma_{R}^{k}$, respectively, assuming that each base is sampled independently of all others. The complementary probability of not getting *k* matches in a row on the forward strand is $1-\Sigma_{F}^{k}$ and, comparably, $1-\Sigma_{R}^{k}$ for the reverse strand. Because $X_{1}$ contains *L* bases, there are a total of L−k+1  *k*-mers in $X_{1}$, so the probability of a *K* not matching at any *k*-mers in $X_{1}$ is approximately $(1-\Sigma_{F}^{k}{)}^{L-k+1}$ and $(1-\Sigma_{R}^{k}{)}^{L-k+1}$. It should be noted that at this step we are assuming that the chance of *K* mismatching at a given position in $X_{1}$ is independent of the chance that *K* mismatches at other positions in $X_{1}$, which is not strictly true. For example, if *K* is the sequence “AAAAA,” and we compare *K* to a subsequence in $X_{1}$ that is “AACAA” we would say that *K* and this subsequence do not match. However, with this information we would also know with certainty that adjacent *k*-mers in $X_{1}$ will also not match *K* because they will still contain a “C.” However, we will continue with assuming that the mismatch between $X_{1}$ and *K* is independent at all positions because this makes our final equations more conservative (decreasing the $\Sigma_{F}^{k}$ term in equation (5) will decrease the overall value of equation (5)).

We can now say that the probability of finding the *k*-mer *K* at least once by chance alone at any of the L−k+1 positions in the sequence $X_{1}$ is:


(3) $$P(K\in X_{1F})=1-{\left(1-\Sigma_{F}^{k}\right)}^{\left(L-k+1\right)}$$

(4) $$P(K\in X_{1R})=1-{\left(1-\Sigma_{R}^{k}\right)}^{\left(L-k+1\right)}.$$

Assuming k<<L, equations (3) and (4) approximate to:


(5) $$P(K\in X_{1F})\approx 1-{\left(1-\Sigma_{F}^{k}\right)}^{L}$$

(6) $$P(K\in X_{1R})\approx 1-{\left(1-\Sigma_{R}^{k}\right)}^{L}.$$

**Fig. 1.**
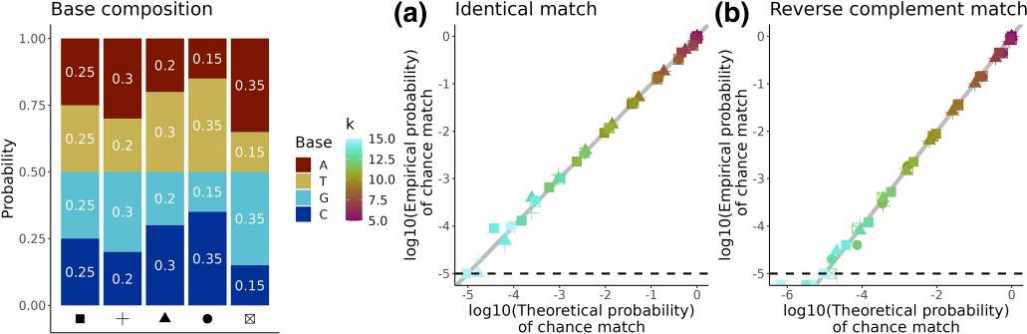
The relationship of *k*-mer length and base composition to chance matching. We verified a) Equation (5) and b) Equation (6) by randomly generating 5.5 million pairs of *k*-mers and genomes. Each simulation had 100,000 trials. For each trial we randomly generated a genome of length 10,000 bases using a given base composition ($p_{A}$, $p_{T}$, $p_{G}$, $p_{C}$ in barchart) and also generated a random *k*-mer (color gradient shows *k*) using the same base composition. We then checked whether the random *k*-mer was in the random genome. In total, the 5 different base compositions × 11 different *k*-mer lengths × 100,000 trials per simulation gives 5.5 million trials total. Grey line is where the theoretical (i.e. Equations (5) and (6)) and empirical probabilities (i.e. from simulations) of a chance match are equal. Dotted black line shows the resolution of the simulations; because we did 100,000 trials per simulation we cannot empirically estimate probabilities smaller than 1/100,000.

We confirmed that equations 5 and (6) work as expected in simulations (Fig. 1). Similar to Fofanov et al. (2004), we can then solve these equations to give the minimum *k*-mer length required to achieve a desired probability of chance *k*-mer matching of $q=P(K\in X_{1F})=P(K\in X_{1R})$:


(7) $$k_{F}=\left\lceil \log_{\Sigma_{F}}\;\left(1-(1-q{)}^{1/L}\right)\right\rceil$$

(8) $$k_{R}=\left\lceil \log_{\Sigma_{R}}\;\left(1-(1-q{)}^{1/L}\right)\right\rceil$$

and now given a choice of *q* we are willing to tolerate, we can then use $\max(k_{F},k_{R})$ as a potential choice of *k*.

**Fig. 2.**
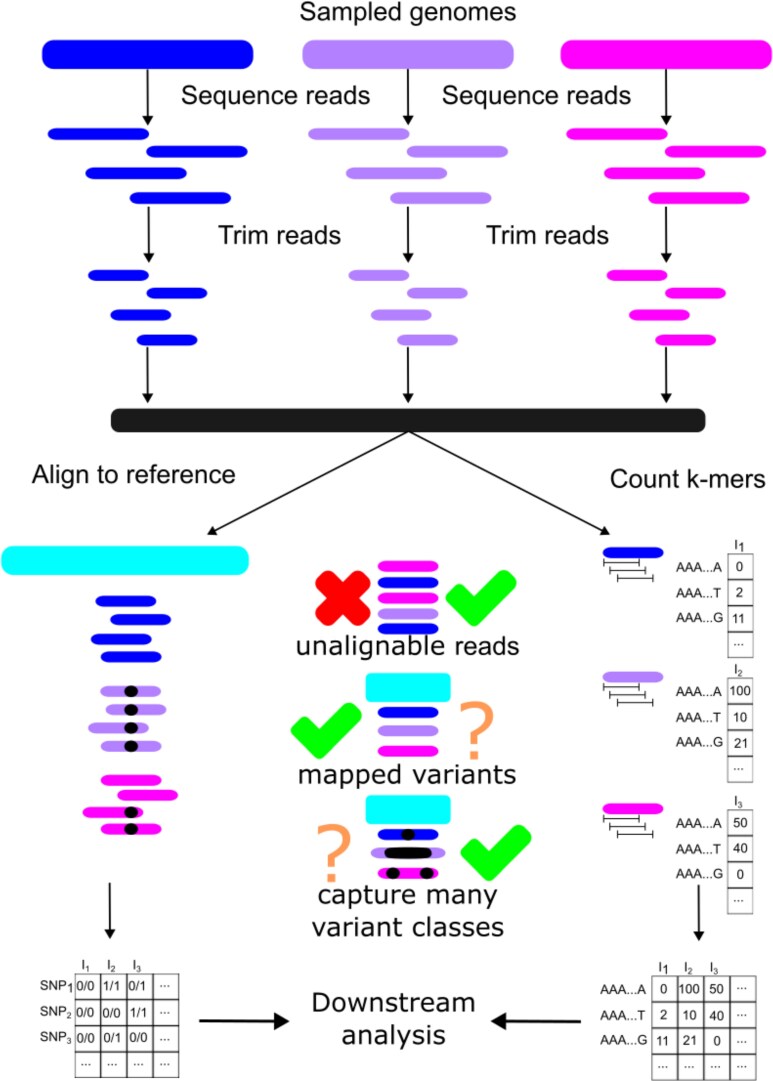
Comparison between a typical SNP-calling workflow and a *k*-mer counting workflow. A typical *k*-mer-based analysis begins the same as a SNP-based analysis: sequencing reads from sampled genomes followed by trimming and other quality control steps on the reads. The crucial difference comes down to whether the remaining reads are aligned to a reference sequence (discarding any unaligned reads) or are used for *k*-mer counting. Workflows may vary in terms of attempting to map *k*-mers to specific loci or calling variants other than SNPs (denoted with “?”). The end result for each workflow is typically a matrix where each row is a different genomic variant, each column is a sample, and the elements represent the variant states. For SNPs, the standard variant call format notation is to use 0/0 to represent homozygous reference genotypes, 0/1 or 1/0 to represent heterozygous genotypes, and 1/1 to represent homozygous alternate genotypes. In *k*-mer-based analyses, the variant states are instead counts of how many *k*-mers of a particular type were observed in each sample.

Equations (7) and (8) demonstrates that k=19 reduces the probability of two 3 Gb genomes of random sequence sharing the same *k*-mer by chance to just 1% (Ondov et al. 2016) (assuming all bases are in equal proportion in both genomes, $p_{A1}=p_{T1}=p_{G1}=p_{C1}=p_{A2}=p_{T2}=p_{G2}=p_{C2}=1/4$) while k=27 gives $q=1\times 10^{-6}$ for a 10 Gb genome where both $X_{1}$ and $X_{2}$ have a GC content of 42% ($p_{A1}=p_{T1}=p_{A2}=$  $p_{T2}=0.29$ and $p_{G2}=p_{C2}=p_{G2}=p_{C2}=0.21$). Slightly longer *k*-mers are required when the proportion of each base is not 25% in both genomes because it is more likely then to have low-complexity strings of bases which make spurious matches more likely. For example, the required *k*-mer length increases to 34 bp if the GC content of both genomes is 21% ($p_{A1}=p_{T1}=p_{A2}=p_{T2}=0.395$ and $p_{G1}=p_{C1}=p_{G2}=p_{C2}=$  0.105), given a 10 Gb genome and $q=1\times 10^{-6}$.

In summary, if *k* is high enough, two genomes in a population are highly unlikely to share *k*-mers just through the accumulation of random sequences alone, suggesting that shared *k*-mers usually reflect shared ancestry. Developing additional *k*-mer selection criteria that explicitly model the influence of shared ancestry on the probability of *k*-mer sharing could improve approaches to choosing *k*.

### Choosing *k* Based on the Balance of Shared vs Differing *k*-mers

A final class of approaches for choosing *k* focuses on how if *k* is small, most *k*-mers are shared between most samples, but as *k* increases more sample-specific *k*-mers occur until no shared *k*-mers remain (Zhang et al. 2017). Neither of these scenarios is usually desirable—the former makes all samples appear to be identical, while the latter makes all samples appear to be completely different. Thus, choosing *k* boils down to balancing the number of shared vs differing *k*-mers. Some statistics for informing this choice include cumulative relative entropy (Sims et al. 2009), relative sequence divergence (Sims et al. 2009), average number of common features (Zhang et al. 2017), Shannon’s diversity index (Zhang et al. 2017), and $\chi^{2}$ tests (Bai et al. 2017). However, there are two main drawbacks of these statistics. First, they were mainly developed for phylogenetic studies focused on short amino acid *k*-mers, so their utility for population genetics (which would probably focus on longer nucleotide *k*-mers) is untested. Furthermore, different values of *k* will appear optimal for genomes of different sizes (Zhang et al. 2017), making it hard to imagine that one optimal *k* exists for a population exhibiting substantial genome size variation. Extending these or similar approaches to optimize *k* for populations with genome size variation will be very useful for future studies.

## Box 2: What’s the Expected Value of *k*-mer Diversity in a Neutrally Evolving Population?

We argue that *k*-mers can be useful for initial assessments of genetic diversity, but two key questions are likely of interest to population geneticists: can *k*-mer diversity be related to nucleotide diversity (the common measure of genetic diversity), and what is the expected value of *k*-mer metrics for populations evolving under a given model? Here, we derive a simple bound on the expected number of *k*-mer differences between a pair of individuals in a neutrally evolving population. Our result is similar to Shi et al. (2024), except we generalize beyond haploid organisms.

We start by assuming that (i) *k* is sufficiently long to capture all of the unique sequences in a genome, (ii) each genome is sequenced at sufficient coverage to confidently identify all of the *k*-mers present, (iii) *k*-mers are sequenced without error, and (iv) only SNPs contribute to differences between genomes. Let *i* and *j* be individuals in a neutrally evolving population with ploidy level *x* and let *y* and *z* be two haploid genomes in the pool of *i* and *j* that differ by origin. Next, let $K_{i}$ and $K_{j}$ represent the set of *k*-mers identified in individual *i* and *j*’s genomes respectively and let $\left|K_{i}\right|$ denote the size of the set $K_{i}$. If all of the genomes within *i* and *j* are identical, then all *k*-mers are shared between them. If we start by assuming *i* and *j* are haploid, this means a maximum of 2k  *k*-mers are not shared between *i* and *j* for every pairwise difference between *i* and *j* (Iqbal et al. 2012; Younsi and MacLean 2015). This relationship can be written as:


(9) $$|K_{i}\cup K_{j}|-|K_{i}\cap K_{j}|\leq 2kD_{yz},$$

where $D_{yz}$ represents the number of pairwise differences between haplotypes *y* and *z* and the left side represents the number of *k*-mers exclusive to either *i* or *j*. The reason for the using a “≤” in equation (9) is to account for three possibilities: (i) if two SNPs are less than *k* bases apart, there will be fewer than 4k  *k*-mers not shared between *i* and *j*, (ii) it is possible for a SNP to turn one *k*-mer into a different *k*-mer that’s already present elsewhere in a genome, and (iii) at higher ploidy levels, if a segregating site is heterozygous in *i* and *j* then *i* and *j* will share all *k*-mers between them.

To generalize beyond haploid organisms, we will replace the conversion factor of 2 in equation (9) above with a general function a(x):


(10) $$|K_{i}\cup K_{j}|-|K_{i}\cap K_{j}|\leq a(x)\sum_{y<z}D_{yz},$$

where a(x) is a conversion factor that turns a number of pairwise differences into a number of sample-exclusive *k*-mers as a function of *x* and $\sum_{y<z}D_{yz}$ is all of the pairwise differences for any pair of haplotypes *y* and *z* in *i* and *j*. The values that maximize a(x), maintaining the validity of using a “≤” sign in Equation (10), are as follows:


(11) $$a(x)=\left\{ \begin{array}{cc} \frac{2k}{x^{2}},\; & \text{if}\;1\leq x\leq 3.\\ \frac{k}{2x-1},\; & \text{if}\;x\geq 4. \end{array}\right.$$

When the population is haploid (x=1), Equation (10) reduces to Equation (9). When x= 2 or 3, a(x) is maximized when *i* and *j* are homozygous for different alleles, creating 2k sample-exclusive *k*-mers for every 4 or 9 pairwise differences, respectively. However, when x≥4, the situation that maximizes a(x) is one where the SNP exists on only one haplotype in *i* or *j*, creating *k* sample-exclusive *k*-mers for every 2x−1 pairwise differences.

Now, we will convert the right side of Equation (10) into nucleotide diversity (*π*). Summing across all pairs of individuals gives:


(12) $$\sum_{i<j}|K_{i}\cup K_{j}|-|K_{i}\cap K_{j}|\leq a(x)\sum_{i<j}\sum_{y<z}D_{yz}.$$

Next, we convert the right-hand side into genome-wide average *π* by dividing both sides by the number of pairwise haplotype comparisons (Korunes and Samuk 2021), which is the number of individuals sampled *n* times ploidy *x*, choose 2:


(13) ∑i<j|Ki∪Kj|−|Ki∩Kj|(nx2)≤a(x)(∑i<j∑y<zDyz(nx2))

(14) ∑i<j|Ki∪Kj|−|Ki∩Kj|(nx2)≤a(x)π.

Next, isolating *π* on one side gives:


(15) ∑i<j|Ki∪Kj|−|Ki∩Kj|a(x)(nx2)≤π.

The intuition behind this formula is that, in a world where our assumptions are met, the number of *k*-mers that are not shared between a pair of samples is bounded by a multiple of *π*. The need to scale *π* by a(x) to get a bound reflects the fact that a given SNP can be captured by multiple *k*-mers, but the exact relationship between pairwise differences and sample-exclusive *k*-mers depends on ploidy.

Finally, we can substitute *π* for the standard formula for the expected value of *π* in a neutrally evolving population at equilibrium (Tajima 1996):


(16) E[∑i<j|Ki∪Kj|−|Ki∩Kj|a(x)(nx2)]≤2xNeμ1+432xNeμ,

where $N_{e}$ is effective population size and *μ* is the mutation rate (probability of mutation per base pair per generation). The denominator on the right-hand side of Equation (16) ensures that the expected value of *π* saturates as it approaches it is theoretical maximum of 0.75 (Tajima 1996). Altogether, equations (15) and (16) provide simple bounds for the average number of *k*-mers that differentiate a pair of samples in terms of $N_{e}$ and *μ*.

## Identifying Variation with *k*-mers

Many of the earlier studies using *k*-mers to identify variants de novo focus on microbes, where (despite small genome sizes) high levels of diversity make alignment and choosing a reference genome difficult (Gardner and Slezak 2010). However, similar approaches have now been extended to other taxa. One class of approaches calls SNPs de novo simply by comparing *k*-mers between samples and searching for pairs of *k*-mers that differ at their central basepair (Gardner and Slezak 2010; Gardner and Hall 2013; Bedo et al. 2016; Li et al. 2022). Another, more popular, class of methods relies on de Bruijn graphs, which are graphs where *k*-mers are nodes and two nodes are connected if they share k−1 bases (Compeau et al. 2011). SNPs in a sample then appear as “bubbles” in these graphs and calling SNPs amounts to searching for these bubbles (Iqbal et al. 2012; Leggett et al. 2013; Uricaru et al. 2015; Younsi and MacLean 2015; Standage et al. 2019; Gauthier et al. 2020). Finally, other approaches first identify *k*-mers that are present in all samples (i.e. “anchor” *k*-mers) and finds paths between anchor *k*-mers through either local alignment or traversing de Bruijn graphs (Audano et al. 2018; Kaplinski et al. 2021; Aylward et al. 2023). The main drawback to these approaches is that the relative positions of the resulting variant calls are usually unknown without further analysis (see Fig. 2).

To call variants with known relative positions, it is possible to combine the strengths of *k*-mers with either pangenomes or databases of previously identified variants. This is because unique variations in pangenomes will often be tagged by multiple unique *k*-mers. For example, one can compare *k*-mers with a reference set of SNPs (Shajii et al. 2016; Pajuste et al. 2017; Denti et al. 2019; Shi et al. 2023; Chu et al. 2024) or insertions (Puurand et al. 2019) to quickly genotype samples without alignment. Or if a reference pangenome assembly is available, it is possible to use *k*-mers to infer the path through the pangenome corresponding to a given sample genome (Iqbal et al. 2012; Ebler et al. 2022; Grytten et al. 2022; Häntze and Horton 2023). However, similar to common alignment-based SNP calling practices, the called variants will be limited to variants present in the pangenome reference.

If references are not already available, *k*-mers can be especially helpful for identifying and assessing variation in species with little prior knowledge. For example, the popular tools genomescope and smudgeplot use the distribution of *k*-mer counts in a sample (i.e. the *k*-mer frequency spectrum) to rapidly estimate important parameters, including genome size, heterozygosity, and ploidy structure all from unassembled read sets (Vurture et al. 2017; Ranallo-Benavidez et al. 2020). *k*-mer frequency spectra can also identify unwanted variation that is potentially due to contamination (reviewed in Cornet and Baurain 2022) or sequencing error. *k*-mers have the useful property that sequencing errors mainly manifest as *k*-mers that occur just once or a few times in a sample of reads (Kelley et al. 2010). This is because random sequencing errors are unlikely to generate the same *k*-mer many times and (if *k* is long enough, see Box 1) usually produce *k*-mers not found in the target genome. Thus, excluding low-copy *k*-mers from an analysis can mitigate sequencing error and one can examine the *k*-mer frequency spectrum to choose an appropriate *k*-mer count cutoff (Zhao et al. 2018). Although *k*-mer frequency spectra can be usefully mined for genome parameters, their exact relationship to key population genetic parameters, such as measures of differentiation or diversity, remains underexplored.

## Measuring Variation with *k*-mers

After calling variants, quantifying levels of variation is a crucial step in many population genetics workflows. Because counting *k*-mers tends to be faster than alignment, *k*-mers could be especially helpful for rapid, initial assessments of diversity that complement or guide pangenome analysis. The standard approach to measure diversity in a population is to align sample sequences to a reference genome then calculate either (i) the average level of heterozygosity across sites or (ii) the number of variants segregating in the sample. These are represented by Nei’s (Equation (17); Nei and Li 1979; Nei and Tajima 1981) and Watterson’s (Equation (18); Watterson 1975) estimators of diversity, respectively:


(17) $$\pi =\left(1-\sum_{i}p_{i}^{2}\right)\left(\frac{n}{n-1}\right)$$

(18) $$\theta_{w}=\frac{S}{\sum_{a=1}^{a=n-1}\frac{1}{a}},$$

where *n* is the number of sequences in the sample, $p_{i}$ is the frequency of the *i*th allele at a locus, and *S* is the number of segregating sites at a locus. While $\theta_{w}$ is based on a discrete count of variants (*S*), *π* is shaped by the allele frequencies of variants and will be higher if variants are common than if variants are rare (note that *π* is more commonly rewritten in terms of the average number of differences between sequences Korunes and Samuk 2021).

Can analogous measures of variation be derived from *k*-mers? There are over 30 valid measures of genetic difference based on *k*-mer counts used in previous literature (Benoit et al. 2016; Zielezinski et al. 2017; Luczak et al. 2019; Zielezinski et al. 2019). However, the three most common *k*-mer dissimilarity measures are arguably Jaccard dissimilarity (Equation (19); Ondov et al. 2016), Bray–Curtis dissimilarity (Equation (20); Dubinkina et al. 2016; Benoit et al. 2020), and cosine dissimilarity (Equation (21), Choi et al. 2019):


(19) $$J(K_{i},K_{j})=1-\frac{K_{i}\cap K_{j}}{K_{i}\cup K_{j}}$$

(20) $$B(C_{i},C_{j})=1-2\sum_{b=1}^{4^{k}}\frac{\min(m_{b}(C_{i}),m_{b}(C_{j}))}{m_{b}(C_{i})+m_{b}(C_{j})}$$

(21) $$C(C_{i},C_{j})=1-\frac{C_{i}\cdot C_{j}}{\left\|C_{i}\|\times \|C_{j}\right\|},$$

where *k* is the length of *k*-mers to be included in the comparison, $K_{i}$ and $K_{j}$ are the set of *k*-mers of length *k* present in a set of reads *i* and *j*, $C_{i}$ and $C_{j}$ are vectors of *k*-mer counts in a set of reads *i* and *j*, and $m_{i}$ is a function that returns the relative frequency of the *b*th *k*-mer in a set (i.e. standardized such that $\sum_{b=1}^{4^{k}}m_{b}(C_{i})$ and $\sum_{b=1}^{4^{k}}m_{b}(C_{j})$ equal 1). These measures work similarly to the classical *π* and $\theta_{w}$ measures: the numerators are a measure of the number of sites that vary between individuals, while the denominators are a measure of sample size (here the number of *k*-mers rather than number of haplotypes). The main difference, however, is that *k*-mer-based measures of genetic dissimilarity are not directly interpretable in terms of mutations, like *π* and $\theta_{w}$ can be with the assumption that each SNP represents one mutation (Haubold et al. 2011; Haubold and Pfaffelhuber 2012). Any given *k*-mer may represent the combined presence of multiple mutations (Voichek and Weigel 2020; Blanca et al. 2022) and any mutation can generate multiple new *k*-mers. Although this means that *k*-mer-based genetic dissimilarity measures are only proxies for the true mutational distance between individuals, they still effectively resolve relationships between lineages compared to alignment-based measures (VanWallendael and Alvarez 2022).

While Equations (19), (20), (21) have all been successfully used to measure genetic dissimilarity between samples in past studies, the formulae highlight their benefits and drawbacks. First, Jaccard dissimilarity, perhaps the most commonly used *k*-mer dissimilarity metric (Ondov et al. 2016; Ruperao et al. 2023), requires only knowing *k*-mer presence/absence patterns in samples instead of *k*-mer counts and thus can take less memory to calculate than other *k*-mer-based dissimilarity measures. However, as a consequence Jaccard dissimilarity may not capture the effects of copy number variation and does not account for variation in coverage between samples, which affects whether a given *k*-mer is called as “present” in a sample (VanWallendael and Alvarez 2022). Approaches that measure *k*-mer counts, like Bray–Curtis dissimilarity (Equation (20)) and cosine dissimilarity (Equation (21)), can better account for these influences, but may require more memory for storing counts (Liu et al. 2017; Choi et al. 2019). To alleviate this memory problem, many approaches calculate approximate *k*-mer dissimilarity measures with a small subset of *k*-mers (Ondov et al. 2016; Zhao 2019; Benoit et al. 2020; Pellegrina et al. 2020). An alternative approach would be to instead compress the *k*-mer counts into a smaller array, keeping information from more *k*-mers while simultaneously alleviating memory burdens (Melsted and Pritchard 2011), but such approaches have not been used to calculate genetic dissimilarity before. The relationship between *k*-mer-based dissimilarity measures and *π* is also rarely explored. While some studies have investigated the relationship between *π* and Jaccard dissimilarity (VanWallendael and Alvarez 2022), other *k*-mer dissimilarity measures are possible and no studies to our knowledge have compared these approaches for populations of varying levels of diversity—a key determinant of whether alignment-based genotype calls are accurate (Cornish and Guda 2015; Bush et al. 2020). In the following sections, we investigate the efficacy of compressed and uncompressed *k*-mer-based measures of variation at capturing the true pairwise diversity of simulated populations.

### Testing the Efficacy of *k*-mer Measures of Variation

We used simulations to investigate the relationship between the true value of *π* and genetic diversity measured from *k*-mer-based approaches from simulated sequencing reads.

#### Simulations

We simulated a neutrally evolving 100kb segment of the *Arabidopsis thaliana* genome. We first forward simulated 300 neutrally evolving populations with SLiM 3 (Haller and Messer 2019). Each population consisted of 100 individuals simulated for 1,000 generations with a uniform recombination rate of $10^{-8}$. To vary the diversity across simulations we varied the mutation rate between $10^{-6}$ and $2*10^{-4}$. For each simulation, we tracked the ancestry through tree-sequence recording which records the geneological history of all samples (Haller et al. 2019). From these trees, we generated sequences based on the *A. thaliana* genome (chromosome 1 at positions 4,185,001–4,285,000) (Kent et al. 2002). We took a sub-sample of 10 individuals from the tips of the trees and randomly assigned nucleotides to each SNP in the sample using msprime version 1.2.0 and tskit version 0.5.6 (Baumdicker et al. 2022). From the sub-tree of the sampled genomes, we recorded the exact number of true average pair-wise differences ($\pi_{t}$) across the sample of 10 individuals (20 chromosomes) using msprime (Baumdicker et al. 2022).

#### Generating *k*-mers

To test the performance of the *k*-mer measures on unaligned reads, we simulated reads for each genome using an Illumina read simulator, InSilicoSeq 2.0.0 (Gourlé et al. 2019). We varied the read count to later investigate the effect of coverage as described below. We generated two sets of reads for each individual at coverages of 10× and 30×. After simulating reads, we counted *k*-mers within the reads using KMC3 (Deorowicz et al. 2015; Kokot et al. 2017). We generated *k*-mer count vectors with k=10,20,30, and 40 for each individual. We used a threshold-based approach to adjust the *k*-mer vectors to reduce the effects of sequencing errors; any *k*-mer count below the threshold value was set to zero for a particular sample before any dissimilarity calculations. For a threshold of 5, only *k*-mers with counts of 5 or more were considered when calculating the difference between two or more groups of *k*-mer counts. We present data with a threshold of 5, but note that a threshold of 0 is qualitatively similar with dissimilarity scores being slightly higher overall. This practice of filtering out low-coverage *k*-mers is analogous to the common practice of filtering out SNPs below a given minor allele frequency threshold (Asif et al. 2021). After *k*-mer counting, we calculated the genetic dissimilarity of populations with the Bray–Curtis (Equation (20)) and cosine dissimilarity (Equation (21)) measures.

#### The Effect of *k* on *k*-mer Similarity Metrics

**Fig. 3.**
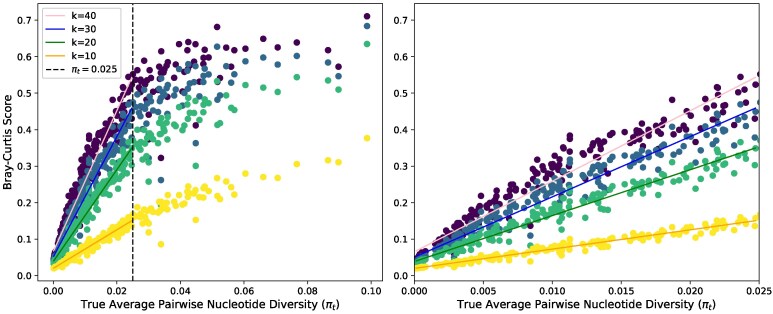
Bray–Curtis dissimilarity calculated from simulated reads with a coverage of 30 where each point represents a sample of 20 chromosomes. Displayed are 10-mers (yellow), 20-mers (green), 30-mers (blue), and 40-mers (purple) each with a linear regression line for $\pi_{t}$ between 0 and 0.025. The scores for populations with a $\pi_{t}$ <0.025 is shown to the right.

Figure 3 shows the effect of *k* on the Bray–Curtis score with 30× coverage. We observe a plateau in the scores when diversity exceeds π≈2.5%. This plateau occurs because, when diversity is high, SNPs cause most of the *k*-mers to be different between two samples. This effect is especially true with high *k* values as one variant sampled in only one read can appear in up to 2k  *k*-mers if it is sampled away from the edges of a read. The elevated number of *k*-mers at high diversity means that it is harder to interpret differences in dissimilarity measures between samples above this threshold, but this problem can be mitigated by using a lower *k* value, such as k=10 when the expected diversity in a sample is high. If $\pi_{t}$ is expected to be below 0.025, larger *k* values can have higher precision and capture more unique *k*-mers in individual samples, better estimating true diversity.

While the data presented in Fig. 3 were simulated with a coverage of 30×, supplementary Figure S1, Supplementary Material online shows that a coverage of 10× results in qualitatively similar scores when k=30. While coverage affects the precision of the measures, the overall trends and relative rankings between simulations remain consistent.

#### The Counting Bloom Filter Approach to Comparing Genomes

**Fig. 4.**
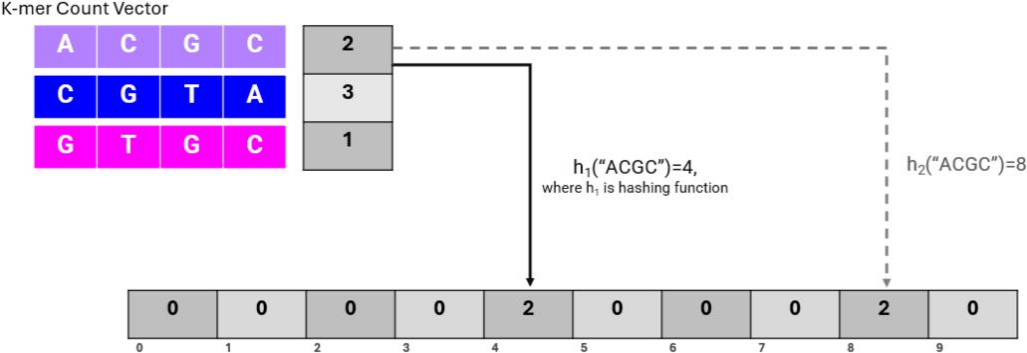
A schematic of a Counting Bloom Filter where the first 4-mer, “ACGC” is hashed. The hash function $h_{1}$ outputs 4 and $h_{2}$ outputs 8. Both positions in the CBF array are incremented by 2 as that is the count of “ACGC” in the given *k*-mer count vector.

Bloom filters are a data structure that can compress a *k*-mer count vector into a smaller array (Melsted and Pritchard 2011). We can compute the dissimilarity of two compressed vectors using less memory and with increased efficiency as fewer entries are compared. Counting bloom filters (CBFs) are modifications of bloom filters (Fan et al. 2000) and are used in alternative *k*-mer count methods and error correction in sequencing data (Shi et al. 2010; Melsted and Pritchard 2011; Roy et al. 2014). CBFs compress the *k*-mer vectors through several hash functions that map to a smaller array. Counting bloom filters will increase the *k*-mer count in the position as opposed to setting the count to one if a *k*-mer has been mapped there (see Fig. 4). Because of the hash-functions and collisions, false positives are possible; here a “false positive” means that two *k*-mers of different sequences may map to the same locations in the vector (i.e. have a hash collision). However, identical mapping of two different *k*-mers is not a concern for relative comparison of diversity, since the same hash functions are used for each sample so collisions are consistent across samples. Collisions will cause diversity to be underestimated, but we can reduce collisions by increasing the number of hash-functions used in the process (Melsted and Pritchard 2011).

**Fig. 5.**
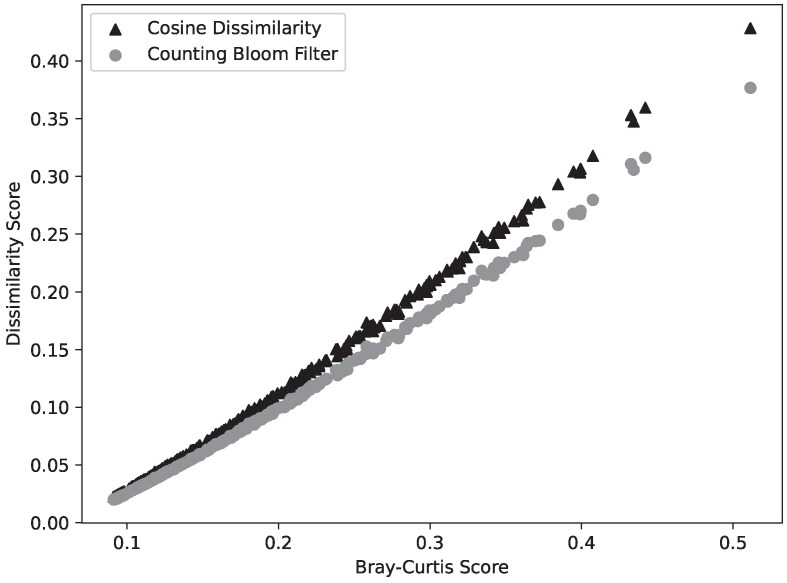
Cosine dissimilarity (triangle) and counting bloom filter scores (circle) from 10-mers of simulated reads with a coverage 30 compared directly against the Bray–Curtis score of each simulated set of 10 individuals (points).

A CBF gives a standardized way to compare across species/experiments if the vectors for *k*-mer counts are set to the same size and the same hash functions are used to generate the vectors across data sets. We can also perform the same cosine dissimilarity measure on the CBF vector that we use directly on the counts of *k*-mers. The cosine dissimilarity measure on the raw *k*-mer count vectors has the same $R^{2}$ to the compressed CBF vectors (0.97) when comparing the scores to $\pi_{t}$. Figure 5 shows that the cosine dissimilarity using the counting bloom filter is qualitatively similar to the cosine dissimilarity with the raw *k*-mer counts. This similarity and the high $R^{2}$ mean that the predictability of $\pi_{t}$ is maintained while using the smaller, more manageable CBF data structure. Therefore, we can reliably use the CBF data structure as opposed to the raw *k*-mer counts to calculate the cosine dissimilarity of two samples.

The memory usage of a *k*-mers vector for a sample with 10× coverage and k=30 under our simulations is around 4.5 MB per sample. The memory usage is reduced to 0.02 MB when compressed to a 10,000 element array of unsigned 16-bit integers. Therefore, storing and using *k*-mer counts for measuring diversity scale much better under the CBF data structure.

#### The Effect of Array Size on CBF Measures of Diversity

We found that the size of the CBF array scales the cosine dissimilarity score and keeps the rank of scores very similar as the array size changes. Since rank is maintained, we can take advantage of much smaller arrays without losing much information about the relative diversity scores between samples. Smaller arrays allow for less memory usage, and faster computing of cosine dissimilarity. Supplementary Figure S2, Supplementary Material online compares the scores of a CBF array of size 20 million and 10 thousand on the same simulations. We observe that the smaller array adjusts the scores down while maintaining the same rank of scores. For example, these results show that if a population has the lowest cosine score with the 20 million length array, it remains among the lowest scores with a 10,000 length array. This result means that it may be possible to adjust the vector size such that the cosine dissimilarity scores are similar in magnitude and scale to the true pairwise nucleotide diversity which can be useful for prediction of the score without alignment, and can make interpretation simpler. However, the viability of this approach still needs to be investigated across different species and genomic contexts, it is likely there is not a “one size fits all” array size that would scale cosine dissimilarity to match *π* universally. Our simulations only consider SNPs, neutral evolution, and the *A. thaliana* genome. It is still left to determine the effects of other mutations types such as insertions, deletions, and inversions as well as other evolution types. Therefore, we should avoid the potential pitfalls of interpreting a scaled value directly as *π* unless future work shows that to be appropriate.

## Explaining Variation with *k*-mers

A common first step to understanding the evolutionary forces shaping variation in populations involves quantifying differentiation between populations. Patterns of differentiation in SNP genotypes are often summarized and plotted using dimension reduction techniques, such as principal component analysis (PCA) (Novembre and Stephens 2008). *k*-mer genotypes are also amenable to PCA and recover the same differentiation patterns as SNP-based PCA (Liu et al. 2017; Murray et al. 2017; Rahman et al. 2018; Ho et al. 2019; Hrytsenko et al. 2022). Applying dimensional reduction to *k*-mers can even differentiate species (Rosen et al. 2008; Aflitos et al. 2015; Bernard et al. 2016; Linard et al. 2019; Boddé et al. 2022), something that can be difficult to do with SNPs without multiple sequence alignment. Interestingly, *k*-mers from repetitive sequences may not always differentiate populations that are clearly differentiated in terms of their SNP genotypes (Renny-Byfield and Baumgarten 2020), suggesting that identifying the set of *k*-mers that best differentiate populations could be an important avenue for future research. Furthermore, while we are aware of one study in yeast that estimates admixture proportions from *k*-mers (Shi et al. 2024), more investigation is needed to see if this approach works for other species.

Besides polymorphism between populations, *k*-mers are also useful for explaining polymorphism patterns across genomes in terms of specific evolutionary forces. For instance, the *k*-mer profile of a given locus is often predictive of the local recombination rate (Liu et al. 2012; Haubold et al. 2013; Haubold 2014; Frenkel et al. 2016; Al Maruf and Shatabda 2019) and the local mutation rate (Aggarwala and Voight 2016; Carlson et al. 2018; Bethune et al. 2022; Adams et al. 2023; Beichman et al. 2023; Liu and Samee 2023). *k*-mers can capture information about recombination and mutation because these processes often associate with specific functional DNA motifs (Myers et al. 2008; Růžička et al. 2017). However, *k*-mers are also intrinsically sensitive to sequence changes and while there are some existing methods to identify new mutations (Nordström et al. 2013; Ho et al. 2019) and sites of recombination (Fletcher et al. 2021) solely from *k*-mers, further development is needed. Being able to predict fine scale variation in mutation and recombination rates solely from the *k*-mer profile of a reference sequence would be extremely beneficial because these processes frequently confound scans for sites of selection (Huber et al. 2016). However, this would require further study of the relationship between *k*-mers and mutation/recombination rates across wider ranges of species.

*k*-mers also offer opportunities to explore patterns of selection. One general assumption in *k*-mer literature is if a *k*-mer is shared across all individuals of a population or species then it is potentially under selection to be conserved (Bernard et al. 2016; Aylward et al. 2023). In contrast, a *k*-mer that is not present in any individual is possibly selected against (although this assumes that *k* is sufficiently small such that the total possible *k*-mer space is small, as might be true when analyzing small amino acid-derived *k*-mers) (Georgakopoulos-Soares et al. 2021). However, it is unlikely that selection is solely responsible for patterns of *k*-mer sharing across individuals or species, so more rigorous approaches are needed. One potentially more rigorous approach would be to investigate *k*-mers that differentiate populations as candidates for loci underlying local adaptation, similar to $F_{ST}$ (Kane and Rieseberg 2007). Although, we are not aware of any papers that employ this specific approach, identifying group-specific *k*-mers is a very common and useful practice, with one example being the assembly of sex-specific sequences (Akagi et al. 2014; Ou et al. 2017; Liao et al. 2020; Neves et al. 2020; Mehrab et al. 2021; Wu et al. 2021; Behrens et al. 2022; Fong et al. 2023; Lichilín et al. 2023). There are some metrics of *k*-mer differentiation in published literature already (Rahman et al. 2018), but they do not have the same interpretation as $F_{ST}$. An alternative approach would be to march through a deBruijn graph (Aylward et al. 2023) to find strings of *k*-mers with selective sweep-like patterns, mainly low diversity, high linkage disequilibrium between *k*-mers, an excess of rare *k*-mers (Alachiotis and Pavlidis 2018), high differentiation between populations (Zhong et al. 2022), and high haplotype homozygosity (Klassmann and Gautier 2022) which could be determined based on *k*-mer copy number (Vurture et al. 2017; Ranallo-Benavidez et al. 2020).

There are only a few model-oriented studies that compare the *k*-mer spectrum observed in a genome to a neutral expectation, mostly in the context of detecting selection on transcription factor binding motifs (Gerland and Hwa 2002; Ke et al. 2008; Raijman et al. 2008; Yeang 2010; Gyorgy 2023). The basic idea behind these approaches is to first derive a neutral substitution model—which describes the probability of one nucleotide being substituted for another in a neutrally evolving sequence (Ke et al. 2008; Raijman et al. 2008)—then measure deviations from that neutral model according to the presence/absence of particular *k*-mers in a genome. Although these models are able to detect selection on *k*-mers that exist in multiple places across a genome, as is the case with binding motifs, it is unclear whether they could be applied to *k*-mers that represent unique genomic sequences.

## Challenges in Investigating *k*-mers

Despite the wide potential of *k*-mers to be useful for population genetic studies, there are three important limitations to keep in mind for most *k*-mer investigations. First, interpreting the biology of specific *k*-mers is often a challenge. One option is to take the candidate *k*-mers identified from an analysis and either align the *k*-mers themselves, the reads containing said *k*-mers, or assembled reads containing said *k*-mers to a reference genome (Voichek and Weigel 2020) or a database of known sequences and motifs. While this is effective at pinpointing concrete type of variants at play in a system, it partially defeats the purpose of using *k*-mers in the first place because *k*-mers of interest may not be present in a reference genome. Second, since each sequencing error can generate up to *k* erroneous *k*-mers and general practice is to discard reads with many unique *k*-mers to reduce the effect of error rates (Zimin et al. 2013), analysis of *k*-mers typically requires datasets with high coverage (>10×) and low-sequencing error rates. These criteria can potentially exclude long-read datasets (Vurture et al. 2017) or reduced-representation datasets where genomes are sequenced at lower coverage to cut costs. Approaches that apply *k*-mers in lower coverage or higher error-rate datasets are needed. Third, *k*-mer-based analyses can frequently involve hundreds of millions or billions of unique *k*-mers. Storing and processing this many *k*-mers at once is often not feasible even on high performance computing systems. Two approaches to solving this issue are to subset one’s *k*-mers (Ondov et al. 2016; Zhao 2019; Benoit et al. 2020; Yi et al. 2021), or, as we discuss here, compress vectors of *k*-mer counts into an array of smaller size (Melsted and Pritchard 2011). However, these solutions are usually developed for specific contexts, such as measuring genomic dissimilarity, and may not be applicable to every population genetic analysis. Future studies need to carefully consider common approaches to decreasing the disk burden of *k*-mer-based analyses and should explore new potential solutions.

These challenges to studying *k*-mers highlight how *k*-mer-based methods should be viewed as complementary to, instead of better than, pangenomes in many situations. Among other benefits, pangenomes have ready biological interpretations and provide greater information on linkage between sequences. However, the fact that *k*-mers can be studied with fewer computing resources and simpler sequencing data sets makes them potentially useful for either preliminary pangenomic analyses or situations where constructing many reference quality genomes is not yet feasible.

## Conclusions

Current literature demonstrates that *k*-mers are useful for identifying, measuring, and explaining variation within populations without performing alignment. Through our own simulations of neutrally evolving populations, we find that *k*-mer dissimilarity reliably scales with nucleotide diversity and *k*-mer matrices can be compressed with minimal loss of dissimilarity information. However, further development is needed to make *k*-mers amenable to a wider array of population genetic tasks, especially the identification of selected loci and population structure. Further developing alignment-free approaches to population genetics tasks will ultimately help guide and complement the analysis of pangenomes.

## Future Directions

- Can we develop criteria for choosing values of *k* that account for population-level variation in genome size or genome content?
- Can we develop *k*-mer-based estimates of population differentiation that are more similar to $F_{ST}$?
- Can we identify putative selective sweeps using *k*-mers?
- Can we develop applications of *k*-mers that are amenable to lower-coverage or pooled sequencing samples that are common in population genetics?
- *k*-mers are useful for getting preliminary estimates of important genome parameters that are useful for tuning pangenome assembly (Ranallo-Benavidez et al. 2020). What other types of preliminary pangenome analyses might *k*-mers be useful for?
- Can *k*-mers, either in combination with or without a pangenome reference, be useful for rapidly estimating allele frequencies and performing demographic inference?

## Supplementary Material

msaf047_Supplementary_Data

## Data Availability

Scripts for simulation and data analysis can be found at: https://github.com/williarj/kmers2024.

## References

[msaf047-B1] Adams CJ, Conery M, Auerbach BJ, Jensen ST, Mathieson I, Voight BF. Regularized sequence-context mutational trees capture variation in mutation rates across the human genome. PLoS Genet. 2023:19(7):e1010807. 10.1371/journal.pgen.1010807. 37418489 PMC10355397

[msaf047-B2] Aflitos SA, Severing E, Sanchez-Perez G, Peters S, de Jong H, de Ridder D. Cnidaria: fast, reference-free clustering of raw and assembled genome and transcriptome NGS data. BMC Bioinformatics. 2015:16(1):352. 10.1186/s12859-015-0806-7. 26525298 PMC4630969

[msaf047-B3] Aggarwala V, Voight BF. An expanded sequence context model broadly explains variability in polymorphism levels across the human genome. Nat Genet. 2016:48(4):349–355. 10.1038/ng.3511. 26878723 PMC4811712

[msaf047-B4] Akagi T, Henry IM, Tao R, Comai L. A Y-chromosome–encoded small RNA acts as a sex determinant in persimmons. Science. 2014:346(6209):646–650. 10.1126/science.1257225. 25359977

[msaf047-B5] Alachiotis N, Pavlidis P. RAiSD detects positive selection based on multiple signatures of a selective sweep and SNP vectors. Commun Biol. 2018:1(1):1–11. 10.1038/s42003-018-0085-8. 30271960 PMC6123745

[msaf047-B6] Al Maruf MA, Shatabda S. iRSpot-SF: prediction of recombination hotspots by incorporating sequence based features into Chou’s Pseudo components. Genomics. 2019:111(4):966–972. 10.1016/j.ygeno.2018.06.003. 29935224

[msaf047-B7] Asif H, Alliey-Rodriguez N, Keedy S, Tamminga CA, Sweeney JA, Pearlson G, Clementz BA, Keshavan MS, Buckley P, Liu C, et al GWAS significance thresholds for deep phenotyping studies can depend upon minor allele frequencies and sample size. Mol Psychiatry. 2021:26(6):2048–2055. 10.1038/s41380-020-0670-3. 32066829 PMC7429341

[msaf047-B8] Audano PA, Ravishankar S, Vannberg FO. Mapping-free variant calling using haplotype reconstruction from *k*-mer frequencies. Bioinformatics. 2018:34(10):1659–1665. 10.1093/bioinformatics/btx753. 29186321 PMC5946877

[msaf047-B9] Aylward AJ, Petrus S, Mamerto A, Hartwick NT, Michael TP. PanKmer: *k*-mer-based and reference-free pangenome analysis. Bioinformatics. 2023:39(10):btad621. 10.1093/bioinformatics/btad621. 37846049 PMC10603592

[msaf047-B10] Bai X, Tang K, Ren J, Waterman M, Sun F. Optimal choice of word length when comparing two Markov sequences using a chi-squared statistic. BMC Genomics. 2017:18(S6):732. 10.1186/s12864-017-4020-z. 28984181 PMC5629589

[msaf047-B11] Baumdicker F, Bisschop G, Goldstein D, Gower G, Ragsdale AP, Tsambos G, Zhu S, Eldon B, Ellerman EC, Galloway JG, et al Efficient ancestry and mutation simulation with msprime 1.0. Genetics. 2022:220(3):iyab229. 10.1093/genetics/iyab229. 34897427 PMC9176297

[msaf047-B12] Becher H, Sampson J, Twyford AD. Measuring the invisible: the sequences causal of genome size differences in eyebrights (Euphrasia) revealed by *k*-mers. Front Plant Sci. 2022:13:1–14. 10.3389/fpls.2022.818410. PMC9372453 35968114

[msaf047-B13] Bedo J, Goudey B, Wazny J, Zhou Z. Information theoretic alignment free variant calling. PeerJ Comput Sci. 2016:2(7283):e71. 10.7717/peerj-cs.71.

[msaf047-B14] Behrens KA, Koblmüller S, Kocher TD. Sex chromosomes in the tribe Cyprichromini (Teleostei: Cichlidae) of Lake Tanganyika. Sci Rep. 2022:12(1):17998. 10.1038/s41598-022-23017-y. 36289404 PMC9606112

[msaf047-B15] Beichman AC, Robinson J, Lin M, Moreno-Estrada A, Nigenda-Morales S, Harris K. Evolution of the mutation spectrum across a mammalian phylogeny. Mol Biol Evol. 2023:40(10):msad213. 10.1093/molbev/msad213. 37770035 PMC10566577

[msaf047-B16] Benoit G, Mariadassou M, Robin S, Schbath S, Peterlongo P, Lemaitre C. SimkaMin: fast and resource frugal de novo comparative metagenomics. Bioinformatics. 2020:36(4):1275–1276. 10.1093/bioinformatics/btz685. 31504187

[msaf047-B17] Benoit G, Peterlongo P, Mariadassou M, Drezen E, Schbath S, Lavenier D, Lemaitre C. Multiple comparative metagenomics using multiset *k*-mer counting. PeerJ Comput Sci. 2016:2(3):e94. 10.7717/peerj-cs.94.

[msaf047-B18] Bernard G, Ragan MA, Chan CX. Recapitulating phylogenies using *k*-mers: from trees to networks. F1000Res. 2016:5:2789. 10.12688/f1000research. 28105314 PMC5224691

[msaf047-B19] Bethune J, Kleppe A, Besenbacher S. A method to build extended sequence context models of point mutations and indels. Nat Commun. 2022:13(1):7884. 10.1038/s41467-022-35596-5. 36550134 PMC9780256

[msaf047-B20] Betschart RO, Thiéry A, Aguilera-Garcia D, Zoche M, Moch H, Twerenbold R, Zeller T, Blankenberg S, Ziegler A. Comparison of calling pipelines for whole genome sequencing: an empirical study demonstrating the importance of mapping and alignment. Sci Rep. 2022:12(1):21502. 10.1038/s41598-022-26181-3. 36513709 PMC9748128

[msaf047-B21] Blanca A, Harris RS, Koslicki D, Medvedev P. The statistics of *k*-mers from a sequence undergoing a simple mutation process without spurious matches. J Comput Biol. 2022:29(2):155–168. 10.1089/cmb.2021.0431. 35108101 PMC11978275

[msaf047-B22] Boddé M, Makunin A, Ayala D, Bouafou L, Diabaté A, Ekpo UF, Kientega M, Le Goff G, Makanga BK, Ngangue MF, et al High-resolution species assignment of *Anopheles* mosquitoes using *k*-mer distances on targeted sequences. Elife. 2022:11:e78775. 10.7554/eLife.78775. 36222650 PMC9648975

[msaf047-B23] Bush SJ, Foster D, Eyre DW, Clark EL, De Maio N, Shaw LP, Stoesser N, Peto TEA, Crook DW, Walker AS. Genomic diversity affects the accuracy of bacterial single-nucleotide polymorphism-calling pipelines. Gigascience. 2020:9(2):giaa007. 10.1093/gigascience/giaa007. 32025702 PMC7002876

[msaf047-B24] Bussi Y, Kapon R, Reich Z. Large-scale *k*-mer-based analysis of the informational properties of genomes, comparative genomics and taxonomy. PLoS One. 2021:16(10):e0258693. REFDOI 10.1371/journal.pone.0258693. 34648558 PMC8516232

[msaf047-B25] Carlson J, Locke AE, Flickinger M, Zawistowski M, Levy S, Myers RM, Boehnke M, Kang HM, Scott LJ, Li JZ, et al Extremely rare variants reveal patterns of germline mutation rate heterogeneity in humans. Nat Commun. 2018:9(1):3753. 10.1038/s41467-018-05936-5. 30218074 PMC6138700

[msaf047-B26] Chen N-C, Solomon B, Mun T, Iyer S, Langmead B. Reference flow: reducing reference bias using multiple population genomes. Genome Biol. 2021:22(1):8. 10.1186/s13059-020-02229-3. 33397413 PMC7780692

[msaf047-B27] Chikhi R, Medvedev P. Informed and automated *k*-mer size selection for genome assembly. Bioinformatics. 2014:30(1):31–37. 10.1093/bioinformatics/btt310. 23732276

[msaf047-B28] Chin C-S, Behera S, Khalak A, Sedlazeck FJ, Sudmant PH, Wagner J, Zook JM. Multiscale analysis of pangenomes enables improved representation of genomic diversity for repetitive and clinically relevant genes. Nat Methods. 2023:20(8):1213–1221. 10.1038/s41592-023-01914-y. 37365340 PMC10406601

[msaf047-B29] Choi I, Ponsero AJ, Bomhoff M, Youens-Clark K, Hartman JH, Hurwitz BL. Libra: scalable *k*-mer–based tool for massive all-vs-all metagenome comparisons. Gigascience. 2019:8(2):giy165. 10.1093/gigascience/giy165. 30597002 PMC6354030

[msaf047-B30] Chu J, Rong J, Feng X, Li H. ntsm: an alignment-free, ultra-low-coverage, sequencing technology agnostic, intraspecies sample comparison tool for sample swap detection. Gigascience. 2024:13:giae024. 10.1093/gigascience/giae024. 38832466 PMC11148594

[msaf047-B31] Compeau PEC, Pevzner PA, Tesler G. Why are de Bruijn graphs useful for genome assembly? Nat Biotechnol. 2011:29(11):987–991. 10.1038/nbt.2023. 22068540 PMC5531759

[msaf047-B32] Cornet L, Baurain D. Contamination detection in genomic data: more is not enough. Genome Biol. 2022:23(1):60. 10.1186/s13059-022-02619-9. 35189924 PMC8862208

[msaf047-B33] Cornish A, Guda C. A comparison of variant calling pipelines using genome in a bottle as a reference. Biomed Res Int. 2015:2015(1):1–11. 10.1155/2015/456479. PMC4619817 26539496

[msaf047-B34] Denti L, Previtali M, Bernardini G, Schönhuth A, Bonizzoni P. MALVA: genotyping by Mapping-free ALlele detection of known VAriants. iScience. 2019:18:20–27. 10.1016/j.isci.2019.07.011. 31352182 PMC6664100

[msaf047-B35] Deorowicz S, Kokot M, Grabowski S, Debudaj-Grabysz A. KMC 2: fast and resource-frugal *k*-mer counting. Bioinformatics. 2015:31(10):1569–1576. 10.1093/bioinformatics/btv022. 25609798

[msaf047-B36] Dubinkina VB, Ischenko DS, Ulyantsev VI, Tyakht AV, Alexeev DG. Assessment of *k*-mer spectrum applicability for metagenomic dissimilarity analysis. BMC Bioinformatics. 2016:17(1):38. 10.1186/s12859-015-0875-7. 26774270 PMC4715287

[msaf047-B37] Durai DA, Schulz MH. Informed kmer selection for de novo transcriptome assembly. Bioinformatics. 2016:32(11):1670–1677. 10.1093/bioinformatics/btw217. 27153653 PMC4892416

[msaf047-B38] Ebler J, Ebert P, Clarke WE, Rausch T, Audano PA, Houwaart T, Mao Y, Korbel JO, Eichler EE, Zody MC, et al Pangenome-based genome inference allows efficient and accurate genotyping across a wide spectrum of variant classes. Nat Genet. 2022:54(4):518–525. 10.1038/s41588-022-01043-w. 35410384 PMC9005351

[msaf047-B39] Fan L, Cao P, Almeida J, Broder A. Summary cache: a scalable wide-area web cache sharing protocol. IEEE ACM Trans Netw. 2000:8(3):281–293. 10.1109/90.851975.

[msaf047-B40] Fletcher K, Zhang L, Gil J, Han R, Cavanaugh K, Michelmore R. AFLAP: assembly-free linkage analysis pipeline using *k*-mers from genome sequencing data. Genome Biol. 2021:22(1):115. 10.1186/s13059-021-02326-x. 33883006 PMC8061198

[msaf047-B41] Fofanov Y, Luo Y, Katili C, Wang J, Belosludtsev Y, Powdrill T, Belapurkar C, Fofanov V, Li T-B, Chumakov S, et al How independent are the appearances of *n*-mers in different genomes? Bioinformatics. 2004:20(15):2421–2428. 10.1093/bioinformatics/bth266. 15087315

[msaf047-B42] Fong LJM, Darolti I, Metzger DCH, Morris J, Lin Y, Sandkam BA, Mank JE. Evolutionary history of the *Poecilia picta* sex chromosomes. Genome Biol Evol. 2023:15(3):evad030. 10.1093/gbe/evad030. 36802329 PMC10003743

[msaf047-B43] Frenkel S, Kirzhner V, Frenkel Z, Korol AB. Organizational heterogeneity of the human genome: significant variation of recombination rate of 100 kbp sequences within GC ranges. In: 2016 Second International Symposium on Stochastic Models in Reliability Engineering, Life Science and Operations Management (SMRLO). 2016. p. 414–420.

[msaf047-B44] Gage JL, Vaillancourt B, Hamilton JP, Manrique-Carpintero NC, Gustafson TJ, Barry K, Lipzen A, Tracy WF, Mikel MA, Kaeppler SM, et al Multiple maize reference genomes impact the identification of variants by genome-wide association study in a diverse inbred panel. Plant Genome. 2019:12(2):180069. 10.3835/plantgenome2018.09.0069. PMC12810008 31290926

[msaf047-B45] Gardner SN, Hall BG. When whole-genome alignments just won’t work: kSNP v2 software for alignment-free SNP discovery and phylogenetics of hundreds of microbial genomes. PLoS One. 2013:8(12):e81760. 10.1371/journal.pone.0081760. 24349125 PMC3857212

[msaf047-B46] Gardner SN, Slezak T. Scalable SNP analyses of 100+ vacterial or viral genomes. J Forensic Res. 2010:1(3):1–5. 10.4172/2157-7145.

[msaf047-B47] Garrison E, Guarracino A, Heumos S, Villani F, Bao Z, Tattini L, Hagmann J, Vorbrugg S, Marco-Sola S, Kubica C, et al Building pangenome graphs. Nat Methods. 2024:21(11):2008–2012. 10.1038/s41592-024-02430-3. 39433878

[msaf047-B48] Gauthier J, Mouden C, Suchan T, Alvarez N, Arrigo N, Riou C, Lemaitre C, Peterlongo P. DiscoSnp-RAD: de novo detection of small variants for RAD-Seq population genomics. PeerJ. 2020:8(2):e9291. 10.7717/peerj.9291. 32566401 PMC7293188

[msaf047-B49] Georgakopoulos-Soares I, Yizhar-Barnea O, Mouratidis I, Hemberg M, Ahituv N. Absent from DNA and protein: genomic characterization of nullomers and nullpeptides across functional categories and evolution. Genome Biol. 2021:22(1):245. 10.1186/s13059-021-02459-z. 34433494 PMC8386077

[msaf047-B50] Gerland U, Hwa T. On the selection and evolution of regulatory DNA motifs. J Mol Evol. 2002:55(4):386–400. 10.1007/s00239-002-2335-z. 12355260

[msaf047-B51] Golicz AA, Bayer PE, Bhalla PL, Batley J, Edwards D. Pangenomics comes of age: from bacteria to plant and animal applications. Trends Genet. 2020:36(2):132–145. 10.1016/j.tig.2019.11.006. 31882191

[msaf047-B52] Gourlé H, Karlsson-Lindsjö O, Hayer J, Bongcam-Rudloff E. Simulating Illumina metagenomic data with InSilicoSeq. Bioinformatics. 2019:35(3):521–522. 10.1093/bioinformatics/bty630. 30016412 PMC6361232

[msaf047-B53] Grytten I, Dagestad Rand K, Sandve GK. KAGE: fast alignment-free graph-based genotyping of SNPs and short indels. Genome Biol. 2022:23(1):209. 10.1186/s13059-022-02771-2. 36195962 PMC9531401

[msaf047-B54] Günther T, Nettelblad C. The presence and impact of reference bias on population genomic studies of prehistoric human populations. PLoS Genet. 2019:15(7):e1008302. 10.1371/journal.pgen.1008302. 31348818 PMC6685638

[msaf047-B55] Gupta PK . GWAS for genetics of complex quantitative traits: genome to pangenome and SNPs to SVs and *k*-mers. Bioessays. 2021:43(11):2100109. 10.1002/bies.v43.11. 34486143

[msaf047-B56] Gyorgy A . Competition and evolutionary selection among core regulatory motifs in gene expression control. Nat Commun. 2023:14(1):8266. 10.1038/s41467-023-43327-7. 38092759 PMC10719253

[msaf047-B57] Haberer G, Kamal N, Bauer E, Gundlach H, Fischer I, Seidel MA, Spannagl M, Marcon C, Ruban A, Urbany C, et al European maize genomes highlight intraspecies variation in repeat and gene content. Nat Genet. 2020:52(9):950–957. 10.1038/s41588-020-0671-9. 32719517 PMC7467862

[msaf047-B58] Hahn MW . Molecular population genetics. Oxford University Press; 2018.

[msaf047-B59] Haller BC, Galloway J, Kelleher J, Messer PW, Ralph PL. Tree-sequence recording in SLiM opens new horizons for forward-time simulation of whole genomes. Mol Ecol Resour. 2019:19(2):552–566. 10.1111/men.2019.19.issue-2. 30565882 PMC6393187

[msaf047-B60] Haller BC, Messer PW. SLiM 3: forward genetic simulations beyond the Wright-Fisher model. Mol Biol Evol. 2019:36(3):632–637. 10.1093/molbev/msy228. 30517680 PMC6389312

[msaf047-B61] Häntze H, Horton P. Effects of spaced *k*-mers on alignment-free genotyping. Bioinformatics. 2023:39(Supplement_1):i213–i221. 10.1093/bioinformatics/btad202. 37387138 PMC10311327

[msaf047-B62] Haubold B . Alignment-free phylogenetics and population genetics. Brief Bioinform. 2014:15(3):407–418. 10.1093/bib/bbt083. 24291823

[msaf047-B63] Haubold B, Krause L, Horn T, Pfaffelhuber P. An alignment-free test for recombination. Bioinformatics. 2013:29(24):3121–3127. 10.1093/bioinformatics/btt550. 24064419 PMC5994939

[msaf047-B64] Haubold B, Pfaffelhuber P. Alignment-free population genomics: an efficient estimator of sequence diversity. G3 (Bethesda). 2012:2(8):883–889. 10.1534/g3.112.002527. 22908037 PMC3411244

[msaf047-B65] Haubold B, Reed FA, Pfaffelhuber P. Alignment-free estimation of nucleotide diversity. Bioinformatics. 2011:27(4):449–455. 10.1093/bioinformatics/btq689. 21156730

[msaf047-B66] Hickey G, Heller D, Monlong J, Sibbesen JA, Sirén J, Eizenga J, Dawson ET, Garrison E, Novak AM, Paten B. Genotyping structural variants in pangenome graphs using the vg toolkit. Genome Biol. 2020:21(1):35. 10.1186/s13059-020-1941-7. 32051000 PMC7017486

[msaf047-B67] Ho EKH, Macrae F, Latt, IV LC, Benner MJ, Sun C, Ebert D, Schaack S. Intraspecific variation in microsatellite mutation profiles in *Daphnia magna*. Mol Biol Evol. 2019:36(9):1942–1954. 10.1093/molbev/msz118. 31077327 PMC6934441

[msaf047-B68] Hrytsenko Y, Daniels NM, Schwartz RS. Determining population structure from *k*-mer frequencies. In: Proceedings of the 13th ACM International Conference on Bioinformatics, Computational Biology and Health Informatics, BCB ’22. New York (NY), USA: Association for Computing Machinery; 2022. p. 1.

[msaf047-B69] Huber CD, DeGiorgio M, Hellmann I, Nielsen R. Detecting recent selective sweeps while controlling for mutation rate and background selection. Mol Ecol. 2016:25(1):142–156. 10.1111/mec.2016.25.issue-1. 26290347 PMC5082542

[msaf047-B70] Iqbal Z, Caccamo M, Turner I, Flicek P, McVean G. De novo assembly and genotyping of variants using colored de Bruijn graphs. Nat Genet. 2012:44(2):226–232. 10.1038/ng.1028. 22231483 PMC3272472

[msaf047-B71] Jaegle B, Pisupati R, Soto-Jiménez LM, Burns R, Rabanal FA, Nordborg M. Extensive sequence duplication in *Arabidopsis* revealed by pseudo-heterozygosity. Genome Biol. 2023:24(1):44. 10.1186/s13059-023-02875-3. 36895055 PMC9999624

[msaf047-B72] Jenike KM, Campos-Domínguez L, Boddé M, Cerca J, Hodson CN, Schatz MC, Jaron KS. *k*-mer approaches for biodiversity genomics. Genome Res. 2025:35(2):219–230. 10.1101/gr.279452.124. 39890468 PMC11874746

[msaf047-B73] Kane NC, Rieseberg LH. Selective sweeps reveal candidate genes for adaptation to drought and salt tolerance in common sunflower, *Helianthus annuus*. Genetics. 2007:175(4):1823–1834. 10.1534/genetics.106.067728. 17237516 PMC1855101

[msaf047-B74] Kaplinski L, Möls M, Puurand T, Pajuste F-D, Remm M. KATK: fast genotyping of rare variants directly from unmapped sequencing reads. Hum Mutat. 2021:42(6):777–786. 10.1002/humu.v42.6. 33715282

[msaf047-B75] Ke S, Zhang XH-F, Chasin LA. Positive selection acting on splicing motifs reflects compensatory evolution. Genome Res. 2008:18(4):533–543. 10.1101/gr.070268.107. 18204002 PMC2279241

[msaf047-B76] Kelley DR, Schatz MC, Salzberg SL. Quake: quality-aware detection and correction of sequencing errors. Genome Biol. 2010:11(11):R116. 10.1186/gb-2010-11-11-r116. 21114842 PMC3156955

[msaf047-B77] Kent WJ, Sugnet CW, Furey TS, Roskin KM, Pringle TH, Zahler AM, Haussler D. The human genome browser at UCSC. Genome Res. 2002:12(6):996–1006. 10.1101/gr.229102. 12045153 PMC186604

[msaf047-B78] Kim J-H, Park J-S, Lee C-Y, Jeong M-G, Xu JL, Choi Y, Jung H-W, Choi H-K. Dissecting seed pigmentation-associated genomic loci and genes by employing dual approaches of reference-based and *k*-mer-based GWAS with 438 *Glycine* accessions. PLoS One. 2020:15(12):e0243085. 10.1371/journal.pone.0243085. 33259564 PMC7707508

[msaf047-B79] Klassmann A, Gautier M. Detecting selection using extended haplotype homozygosity (EHH)-based statistics in unphased or unpolarized data. PLoS One. 2022:17(1):e0262024. 10.1371/journal.pone.0262024. 35041674 PMC8765611

[msaf047-B80] Kokot M, Długosz M, Deorowicz S. KMC 3: counting and manipulating *k*-mer statistics. Bioinformatics. 2017:33(17):2759–2761. 10.1093/bioinformatics/btx304. 28472236

[msaf047-B81] Kolář F, Čertner M, Suda J, Schönswetter P, Husband BC. Mixed-ploidy species: progress and opportunities in polyploid research. Trends Plant Sci. 2017:22(12):1041–1055. 10.1016/j.tplants.2017.09.011. 29054346

[msaf047-B82] Kolekar P, Kale M, Kulkarni-Kale U. Alignment-free distance measure based on return time distribution for sequence analysis: applications to clustering, molecular phylogeny and subtyping. Mol Phylogenet Evol. 2012:65(2):510–522. 10.1016/j.ympev.2012.07.003. 22820020

[msaf047-B83] Korunes KL, Samuk K. pixy: unbiased estimation of nucleotide diversity and divergence in the presence of missing data. Mol Ecol Resour. 2021:21(4):1359–1368. 10.1111/men.v21.4. 33453139 PMC8044049

[msaf047-B84] Leggett RM, Ramirez-Gonzalez RH, Verweij W, Kawashima CG, Iqbal Z, Jones JDG, Caccamo M, MacLean D. Identifying and classifying trait linked polymorphisms in non-reference species by walking coloured de Bruijn graphs. PLoS One. 2013:8(3):e60058. 10.1371/journal.pone.0060058. 23536903 PMC3607606

[msaf047-B85] Lei L, Goltsman E, Goodstein D, Wu GA, Rokhsar DS, Vogel JP. Plant pan-genomics comes of age. Annu Rev Plant Biol. 2021:72(1):411–435. 10.1146/arplant.2021.72.issue-1. 33848428

[msaf047-B86] Lemane T, Chikhi R, Peterlongo P. kmdiff, large-scale and user-friendly differential *k*-mer analyses. Bioinformatics. 2022:38(24):5443–5445. 10.1093/bioinformatics/btac689. 36315078 PMC9750116

[msaf047-B87] Li H . Toward better understanding of artifacts in variant calling from high-coverage samples. Bioinformatics. 2014:30(20):2843–2851. 10.1093/bioinformatics/btu356. 24974202 PMC4271055

[msaf047-B88] Li Y, Patel H, Lin Y. Kmer2SNP: reference-free heterozygous SNP calling using *k*-mer frequency distributions. In: Ng C, Piscuoglio S, editors. Variant calling: methods and protocols. New York (NY): Springer US; 2022. p. 257–265. 10.1007/978-1-0716-2293-3_16 35751820

[msaf047-B89] Liao Q, Du R, Gou J, Guo L, Shen H, Liu H, Nguyen JK, Ming R, Yin T, Huang S, et al The genomic architecture of the sex-determining region and sex-related metabolic variation in Ginkgobiloba. Plant J. 2020:104(5):1399–1409. 10.1111/tpj.v104.5. 33015884

[msaf047-B90] Liao W-W, Asri M, Ebler J, Doerr D, Haukness M, Hickey G, Lu S, Lucas JK, Monlong J, Abel HJ, et al A draft human pangenome reference. Nature. 2023:617(7960):312–324. 10.1038/s41586-023-05896-x. 37165242 PMC10172123

[msaf047-B91] Lichilín N, Salzburger W, Böhne A. No evidence for sex chromosomes in natural populations of the cichlid fish *Astatotilapia burtoni*. G3 (Bethesda). 2023:13(3):jkad011. 10.1093/g3journal/jkad011. 36649174 PMC9997565

[msaf047-B92] Linard B, Swenson K, Pardi F. Rapid alignment-free phylogenetic identification of metagenomic sequences. Bioinformatics. 2019:35(18):3303–3312. 10.1093/bioinformatics/btz068. 30698645

[msaf047-B93] Liu G, Liu J, Cui X, Cai L. Sequence-dependent prediction of recombination hotspots in *Saccharomyces cerevisiae*. J Theor Biol. 2012:293:49–54. 10.1016/j.jtbi.2011.10.004. 22016025

[msaf047-B94] Liu S, Zheng J, Migeon P, Ren J, Hu Y, He C, Liu H, Fu J, White FF, Toomajian C, et al Unbiased *k*-mer analysis reveals changes in copy number of highly repetitive sequences during maize domestication and improvement. Sci Rep. 2017:7(1):42444. 10.1038/srep42444. 28186206 PMC5301235

[msaf047-B95] Liu Z, Samee M. Structural underpinnings of mutation rate variations in the human genome. Nucleic Acids Res. 2023:51(14):7184–7197. 10.1093/nar/gkad551. 37395403 PMC10415140

[msaf047-B96] Luczak BB, James BT, Girgis HZ. A survey and evaluations of histogram-based statistics in alignment-free sequence comparison. Brief Bioinform. 2019:20(4):1222–1237. 10.1093/bib/bbx161. 29220512 PMC6781583

[msaf047-B97] McClelland M . Selection against dam methylation sites in the genomes of DNA of enterobacteriophages. J Mol Evol. 1985:21(4):317–322. 10.1007/BF02115649. 6443311

[msaf047-B98] Mehrab Z, Mobin J, Tahmid IA, Rahman A. Efficient association mapping from *k*-mers–an application in finding sex-specific sequences. PLoS One. 2021:16(1):e0245058. 10.1371/journal.pone.0245058. 33411830 PMC7790365

[msaf047-B99] Melsted P, Pritchard JK. Efficient counting of *k*-mers in DNA sequences using a bloom filter. BMC Bioinformatics. 2011:12(1):333. 10.1186/1471-2105-12-333. 21831268 PMC3166945

[msaf047-B100] Murray KD, Webers C, Ong CS, Borevitz J, Warthmann N. kWIP: the *k*-mer weighted inner product, a de novo estimator of genetic similarity. PLoS Comput Biol. 2017:13(9):e1005727. 10.1371/journal.pcbi.1005727. 28873405 PMC5600398

[msaf047-B101] Myers S, Freeman C, Auton A, Donnelly P, McVean G. A common sequence motif associated with recombination hot spots and genome instability in humans. Nat Genet. 2008:40(9):1124–1129. 10.1038/ng.213. 19165926

[msaf047-B102] Nei M, Li WH. Mathematical model for studying genetic variation in terms of restriction endonucleases. Proc Natl Acad Sci U S A. 1979:76(10):5269–5273. 10.1073/pnas.76.10.5269. 291943 PMC413122

[msaf047-B103] Nei M, Tajima F. DNA polymorphism detectable by restriction endonucleases. Genetics. 1981:97(1):145–163. 10.1093/genetics/97.1.145. 6266912 PMC1214380

[msaf047-B104] Neves CJ, Matzrafi M, Thiele M, Lorant A, Mesgaran MB, Stetter MG. Male linked genomic region determines sex in dioecious *Amaranthus palmeri*. J Hered. 2020:111(7):606–612. 10.1093/jhered/esaa047. 33340320 PMC7846199

[msaf047-B105] Nordström KJV, Albani MC, James GV, Gutjahr C, Hartwig B, Turck F, Paszkowski U, Coupland G, Schneeberger K. Mutation identification by direct comparison of whole-genome sequencing data from mutant and wild-type individuals using *k*-mers. Nat Biotechnol. 2013:31(4):325–330. 10.1038/nbt.2515. 23475072

[msaf047-B106] Novembre J, Stephens M. Interpreting principal component analyses of spatial population genetic variation. Nat Genet. 2008:40(5):646–649. 10.1038/ng.139. 18425127 PMC3989108

[msaf047-B107] Ondov BD, Treangen TJ, Melsted P, Mallonee AB, Bergman NH, Koren S, Phillippy AM. Mash: fast genome and metagenome distance estimation using MinHash. Genome Biol. 2016:17(1):132. 10.1186/s13059-016-0997-x. 27323842 PMC4915045

[msaf047-B108] Onetto CA, Sosnowski MR, Heuvel SVD, Borneman AR. Population genomics of the grapevine pathogen *Eutypa lata* reveals evidence for population expansion and intraspecific differences in secondary metabolite gene clusters. PLoS Genet. 2022:18(4):e1010153. 10.1371/journal.pgen.1010153. 35363788 PMC9007359

[msaf047-B109] O’Rawe J, Jiang T, Sun G, Wu Y, Wang W, Hu J, Bodily P, Tian L, Hakonarson H, Johnson WE, et al Low concordance of multiple variant-calling pipelines: practical implications for exome and genome sequencing. Genome Med. 2013:5(3):28. 10.1186/gm432. 23537139 PMC3706896

[msaf047-B110] Ou M, Yang C, Luo Q, Huang R, Zhang A-D, Liao L-J, Li Y-M, He L-B, Zhu Z-Y, Chen K-C, et al An NGS-based approach for the identification of sex-specific markers in snakehead (*Channa argus*). Oncotarget. 2017:8(58):98733–98744. 10.18632/oncotarget.v8i58. 29228723 PMC5716763

[msaf047-B111] Pajuste F-D, Kaplinski L, Möls M, Puurand T, Lepamets M, Remm M. FastGT: an alignment-free method for calling common SNVs directly from raw sequencing reads. Sci Rep. 2017:7(1):2537. 10.1038/s41598-017-02487-5. 28566690 PMC5451431

[msaf047-B112] Pellegrina L, Pizzi C, Vandin F. Fast approximation of frequent *k*-mers and applications to metagenomics. J Comput Biol. 2020:27(4):534–549. 10.1089/cmb.2019.0314. 31891535

[msaf047-B113] Ponsero AJ, Miller M, Hurwitz BL. Comparison of *k*-mer-based *de novo* comparative metagenomic tools and approaches. Microbiome Res Rep. 2023:2(4):1–21. 10.20517/mrr.2023.26. 38058765 PMC10696585

[msaf047-B114] Puurand T, Kukuškina V, Pajuste F-D, Remm M. AluMine: alignment-free method for the discovery of polymorphic Alu element insertions. Mob DNA. 2019:10(1):31. 10.1186/s13100-019-0174-3. 31360240 PMC6639938

[msaf047-B115] Rahman A, Hallgrímsdóttir I, Eisen M, Pachter L. Association mapping from sequencing reads using *k*-mers. Elife. 2018:7:e32920. 10.7554/eLife.32920. 29897334 PMC6044908

[msaf047-B116] Raijman D, Shamir R, Tanay A. Evolution and selection in yeast promoters: analyzing the combined effect of diverse transcription factor binding sites. PLoS Comput Biol. 2008:4(1):e7. 10.1371/journal.pcbi.0040007. 18193940 PMC2186363

[msaf047-B117] Ranallo-Benavidez TR, Jaron KS, Schatz MC. GenomeScope 2.0 and Smudgeplot for reference-free profiling of polyploid genomes. Nat Commun. 2020:11(1):1432. 10.1038/s41467-020-14998-3. 32188846 PMC7080791

[msaf047-B118] Renny-Byfield S, Baumgarten A. Repetitive DNA content in the maize genome is uncoupled from population stratification at SNP loci. BMC Genomics. 2020:21(1):1–10. 10.1186/s12864-020-6517-0. PMC6993463 32000670

[msaf047-B119] Rice ES, Alberdi A, Alfieri J, Athrey G, Balacco JR, Bardou P, Blackmon H, Charles M, Cheng HH, Fedrigo O, et al A pangenome graph reference of 30 chicken genomes allows genotyping of large and complex structural variants. BMC Biol. 2023:21(1):267. 10.1186/s12915-023-01758-0. 37993882 PMC10664547

[msaf047-B120] Rosen G, Garbarine E, Caseiro D, Polikar R, Sokhansanj B. Metagenome fragment classification using *n*-mer frequency profiles. Adv Bioinformatics. 2008:2008(1):1–12. 10.1155/abi.v2008.1. PMC2777009 19956701

[msaf047-B121] Roy RS, Bhattacharya D, Schliep A. Turtle: identifying frequent *k*-mers with cache-efficient algorithms. Bioinformatics. 2014:30(14):1950–1957. 10.1093/bioinformatics/btu132. 24618471

[msaf047-B122] Ruperao P, Gandham P, Odeny DA, Mayes S, Selvanayagam S, Thirunavukkarasu N, Das RR, Srikanda M, Gandhi H, Habyarimana E, et al Exploring the sorghum race level diversity utilizing 272 sorghum accessions genomic resources. Front Plant Sci. 2023:14:1–15. 10.3389/fpls.2023.1143512. PMC10063887 37008459

[msaf047-B123] Růžička M, Kulhánek P, Radová L, Čechová A, Špačková N, Fajkusová L, Réblová K. DNA mutation motifs in the genes associated with inherited diseases. PLoS One. 2017:12(8):e0182377. 10.1371/journal.pone.0182377. 28767725 PMC5540541

[msaf047-B124] Schmuths H, Meister A, Horres R, Bachmann K. Genome size variation among accessions of *Arabidopsis thaliana*. Ann Bot. 2004:93(3):317–321. 10.1093/aob/mch037. 14724121 PMC4242198

[msaf047-B125] Shajii A, Yorukoglu D, William Yu Y, Berger B. Fast genotyping of known SNPs through approximate *k*-mer matching. Bioinformatics. 2016:32(17):i538–i544. 10.1093/bioinformatics/btw460. 27587672 PMC5013917

[msaf047-B126] Shannon CE . A mathematical theory of communication. Bell Syst Tech J. 1948:27(3):379–423. 10.1002/bltj.1948.27.issue-3.

[msaf047-B127] Shi G, Dai Y, Zhou D, Chen M, Zhang J, Bi Y, Liu S, Wu Q. An alignment- and reference-free strategy using *k*-mer present pattern for population genomic analyses. Mycology. 2024:16(1):309–323. 10.1080/21501203.2024.2358868. 40083414 PMC11899203

[msaf047-B128] Shi H, Schmidt B, Liu W, Müller-Wittig W. Quality-score guided error correction for short-read sequencing data using CUDA. Procedia Comput Sci. 2010:1(1):1129–1138. 10.1016/j.procs.2010.04.125.

[msaf047-B129] Shi ZJ, Nayfach S, Pollard KS. Identifying species-specific *k*-mers for fast and accurate metagenotyping with Maast and GT-Pro. STAR Protoc. 2023:4(1):101964. 10.1016/j.xpro.2022.101964. 36856771 PMC10037184

[msaf047-B130] Sims GE, Jun S-R, Wu GA, Kim S-H. Alignment-free genome comparison with feature frequency profiles (FFP) and optimal resolutions. Proc Natl Acad Sci U S A. 2009:106(8):2677–2682. 10.1073/pnas.0813249106. 19188606 PMC2634796

[msaf047-B131] Sirén J, Monlong J, Chang X, Novak AM, Eizenga JM, Markello C, Sibbesen JA, Hickey G, Chang P-C, Carroll A, et al Pangenomics enables genotyping of known structural variants in 5202 diverse genomes. Science. 2021:374(6574):abg8871. 10.1126/science.abg8871. 34914532 PMC9365333

[msaf047-B132] Song B, Buckler ES, Stitzer MC. New whole-genome alignment tools are needed for tapping into plant diversity. Trends Plant Sci. 2024:29(3):355–369. 10.1016/j.tplants.2023.08.013. 37749022

[msaf047-B133] Song B, Marco-Sola S, Moreto M, Johnson L, Buckler ES, Stitzer MC. AnchorWave: sensitive alignment of genomes with high sequence diversity, extensive structural polymorphism, and whole-genome duplication. Proc Natl Acad Sci U S A. 2022:119(1):e2113075119. 10.1073/pnas.2113075119. 34934012 PMC8740769

[msaf047-B134] Song J-M, Guan Z, Hu J, Guo C, Yang Z, Wang S, Liu D, Wang B, Lu S, Zhou R, et al Eight high-quality genomes reveal pan-genome architecture and ecotype differentiation of *Brassica napus*. Nat Plants. 2020:6(1):34–45. 10.1038/s41477-019-0577-7. 31932676 PMC6965005

[msaf047-B135] Sopniewski J, Catullo RA. Estimates of heterozygosity from single nucleotide polymorphism markers are context-dependent and often wrong. Mol Ecol Resour. 2024:24(4):e13947. 10.1111/men.v24.4. 38433491

[msaf047-B136] Standage DS, Brown CT, Hormozdiari F. Kevlar: a mapping-free framework for accurate discovery of *de novo* variants. iScience. 2019:18:28–36. 10.1016/j.isci.2019.07.032. 31377530 PMC6682328

[msaf047-B137] Tajima F . The amount of DNA polymorphism maintained in a finite population when the neutral mutation rate varies among sites. Genetics. 1996:143(3):1457–1465. 10.1093/genetics/143.3.1457. 8807315 PMC1207412

[msaf047-B138] Uricaru R, Rizk G, Lacroix V, Quillery E, Plantard O, Chikhi R, Lemaitre C, Peterlongo P. Reference-free detection of isolated SNPs. Nucleic Acids Res. 2015:43(2):e11. 10.1093/nar/gku1187. 25404127 PMC4333369

[msaf047-B139] VanWallendael A, Alvarez M. Alignment-free methods for polyploid genomes: quick and reliable genetic distance estimation. Mol Ecol Resour. 2022:22(2):612–622. 10.1111/men.v22.2. 34478242

[msaf047-B140] Venter JC, Adams MD, Myers EW, Li PW, Mural RJ, Sutton GG, Smith HO, Yandell M, Evans CA, Holt RA, et al The sequence of the human genome. Science. 2001:291(5507):1304–1351. 10.1126/science.1058040. 11181995

[msaf047-B141] Voichek Y, Weigel D. Identifying genetic variants underlying phenotypic variation in plants without complete genomes. Nat Genet. 2020:52(5):534–540. 10.1038/s41588-020-0612-7. 32284578 PMC7610390

[msaf047-B142] Vurture GW, Sedlazeck FJ, Nattestad M, Underwood CJ, Fang H, Gurtowski J, Schatz MC. GenomeScope: fast reference-free genome profiling from short reads. Bioinformatics. 2017:33(14):2202–2204. 10.1093/bioinformatics/btx153. 28369201 PMC5870704

[msaf047-B143] Watterson GA . On the number of segregating sites in genetical models without recombination. Theor Popul Biol. 1975:7(2):256–276. 10.1016/0040-5809(75)90020-9. 1145509

[msaf047-B144] Wong KHY, Ma W, Wei C-Y, Yeh E-C, Lin W-J, Wang EHF, Su J-P, Hsieh F-J, Kao H-J, Chen H-H, et al Towards a reference genome that captures global genetic diversity. Nat Commun. 2020:11(1):5482. 10.1038/s41467-020-19311-w. 33127893 PMC7599213

[msaf047-B145] Wu DY, Ugozzoli L, Pal BK, Qian J, Wallace RB. The effect of temperature and oligonucleotide primer length on the specificity and efficiency of amplification by the polymerase chain reaction. DNA Cell Biol. 1991:10(3):233–238. 10.1089/dna.1991.10.233. 2012681

[msaf047-B146] Wu M, Haak DC, Anderson GJ, Hahn MW, Moyle LC, Guerrero RF. Inferring the genetic basis of sex determination from the genome of a dioecious nightshade. Mol Biol Evol. 2021:38(7):2946–2957. 10.1093/molbev/msab089. 33769517 PMC8233512

[msaf047-B147] Yeang C-H . Quantifying the strength of natural selection of a motif sequence. In: Moulton V, Singh M, editors. Algorithms in bioinformatics, Lecture Notes in Computer Science. Berlin, Heidelberg: Springer; 2010. p. 362–373.

[msaf047-B148] Yi H, Lin Y, Lin C, Jin W. Kssd: sequence dimensionality reduction by *k*-mer substring space sampling enables real-time large-scale datasets analysis. Genome Biol. 2021:22(1):84. 10.1186/s13059-021-02303-4. 33726811 PMC7962209

[msaf047-B149] Younsi R, MacLean D. Using 2*k*+2 bubble searches to find single nucleotide polymorphisms in *k*-mer graphs. Bioinformatics. 2015:31(5):642–646. 10.1093/bioinformatics/btu706. 25344498 PMC4341063

[msaf047-B150] Zhang Q, Jun S-R, Leuze M, Ussery D, Nookaew I. Viral phylogenomics using an alignment-free method: a three-step approach to determine optimal length of *k*-mer. Sci Rep. 2017:7(1):40712. 10.1038/srep40712. 28102365 PMC5244389

[msaf047-B151] Zhao L, Xie J, Bai L, Chen W, Wang M, Zhang Z, Wang Y, Zhao Z, Li J. Mining statistically-solid *k*-mers for accurate NGS error correction. BMC Genomics. 2018:19(10):912. 10.1186/s12864-018-5272-y. 30598110 PMC6311904

[msaf047-B152] Zhao X . BinDash, software for fast genome distance estimation on a typical personal laptop. Bioinformatics. 2019:35(4):671–673. 10.1093/bioinformatics/bty651. 30052763

[msaf047-B153] Zhong L, Zhu Y, Olsen KM. Hard versus soft selective sweeps during domestication and improvement in soybean. Mol Ecol. 2022:31(11):3137–3153. 10.1111/mec.v31.11. 35366022

[msaf047-B154] Zhou Y, Zhang Z, Bao Z, Li H, Lyu Y, Zan Y, Wu Y, Cheng L, Fang Y, Wu K, et al Graph pangenome captures missing heritability and empowers tomato breeding. Nature. 2022:606(7914):527–534. 10.1038/s41586-022-04808-9. 35676474 PMC9200638

[msaf047-B155] Zielezinski A, Girgis HZ, Bernard G, Leimeister C-A, Tang K, Dencker T, Lau AK, Röhling S, Choi JJ, Waterman MS, et al Benchmarking of alignment-free sequence comparison methods. Genome Biol. 2019:20(1):144. 10.1186/s13059-019-1755-7. 31345254 PMC6659240

[msaf047-B156] Zielezinski A, Vinga S, Almeida J, Karlowski WM. Alignment-free sequence comparison: benefits, applications, and tools. Genome Biol. 2017:18(1):186. 10.1186/s13059-017-1319-7. 28974235 PMC5627421

[msaf047-B157] Zimin AV, Marçais G, Puiu D, Roberts M, Salzberg SL, Yorke JA. The MaSuRCA genome assembler. Bioinformatics. 2013:29(21):2669–2677. 10.1093/bioinformatics/btt476. 23990416 PMC3799473

