# Supplementary material for: K-mer-based Approaches to Bridging Pangenomics and Population Genetics
Source: Mol Biol Evol. 2025 Mar 5;42(3):msaf047. doi: 10.1093/molbev/msaf047 (PMC11925024; doi:10.1093/molbev/msaf047)
Supplement: msaf047_Supplementary_Data [file msaf047_supplementary_data.pdf]

Supplementary material

The effect of coverage on k-mer measures

We observe in Figure S1 a complex interaction between coverage and the resulting Bray-Curtis score depending on the length of k. With 30-mers, there is very little difference in the Bray-Curtis score. However, 10-mers are more affected by changes in coverage. The overall trend and ranks of the scores remain, but the Bray-Curtis score is generally higher with 10x coverage.

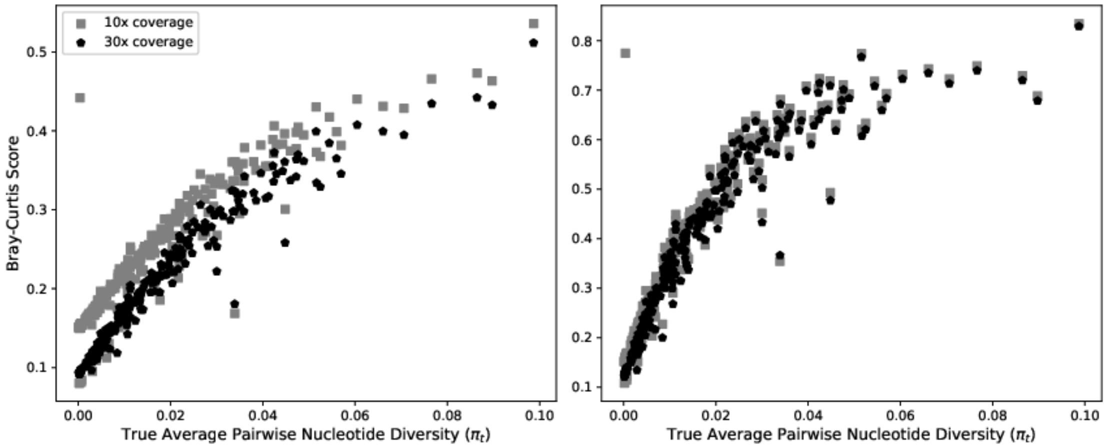

Figure S1: Bray-Curtis scores calculated with 10-mers (left) and 30-mers (right). For each sample, reads with 10x coverage (gray/square) and 30x coverage (black/pentagon) were simulated. After simulating reads, the k-mers were counted and then the Bray-Curtis score is calculated. Each point represents one sample with a specific k and coverage.

Adjusting array size of Counting Bloom Filter

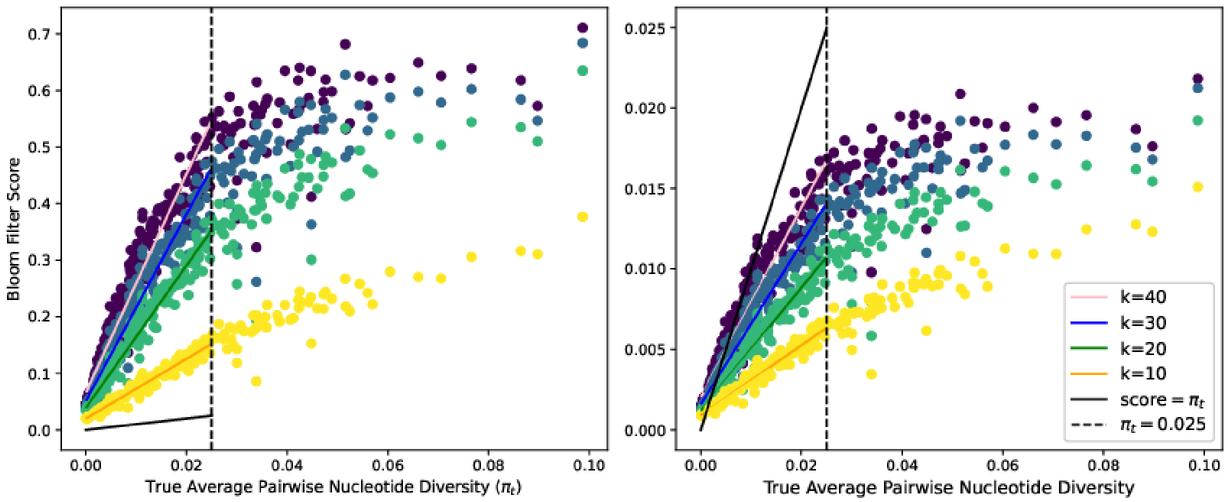

Figure S2: A comparison of cosine similarity scores of CBFs with an array size of 20 million (left) and 10,000 (right). The black line shows the 1-to-1 mapping of π_t to the dissimilarity score. Note the difference in the scales of the scores on the y-axes, and that the right panel shows points that are closer to the 1-to-1 line.
